# Metabolic engineering of *Saccharomyces cerevisiae* for chelerythrine biosynthesis

**DOI:** 10.1186/s12934-024-02448-4

**Published:** 2024-06-21

**Authors:** Jiawei Zhu, Kai Zhang, Yuanzhi He, Qi Zhang, Yanpeng Ran, Zaigao Tan, Li Cui, Yan Feng

**Affiliations:** State Key Laboratory of Microbial Metabolism, School of Life Sciences and Biotechnology, Shanghai Jiao Tong University, 800 Dongchuan Rd, Shanghai, 200240 China

**Keywords:** Chelerythrine, Metabolic engineering, Synthetic biology, Natural product biosynthesis, *Saccharomyces cerevisiae*, Microbial cell factory

## Abstract

**Background:**

Chelerythrine is an important alkaloid used in agriculture and medicine. However, its structural complexity and low abundance in nature hampers either bulk chemical synthesis or extraction from plants. Here, we reconstructed and optimized the complete biosynthesis pathway for chelerythrine from (*S*)-reticuline in *Saccharomyces cerevisiae* using genetic reprogramming.

**Results:**

The first-generation strain Z4 capable of producing chelerythrine was obtained via heterologous expression of seven plant-derived enzymes (McoBBE, TfSMT, AmTDC, EcTNMT, PsMSH, EcP6H, and PsCPR) in *S. cerevisiae* W303-1 A. When this strain was cultured in the synthetic complete (SC) medium supplemented with 100 µM of (*S*)-reticuline for 10 days, it produced up to 0.34 µg/L chelerythrine. Furthermore, efficient metabolic engineering was performed by integrating multiple-copy rate-limiting genes (*TfSMT*, *AmTDC*, *EcTNMT*, *PsMSH*, *EcP6H*, *PsCPR*, *INO2*, and *AtATR1*), tailoring the heme and NADPH engineering, and engineering product trafficking by heterologous expression of *MtABCG10* to enhance the metabolic flux of chelerythrine biosynthesis, leading to a nearly 900-fold increase in chelerythrine production. Combined with the cultivation process, chelerythrine was obtained at a titer of 12.61 mg per liter in a 0.5 L bioreactor, which is over 37,000-fold higher than that of the first-generation recombinant strain.

**Conclusions:**

This is the first heterologous reconstruction of the plant-derived pathway to produce chelerythrine in a yeast cell factory. Applying a combinatorial engineering strategy has significantly improved the chelerythrine yield in yeast and is a promising approach for synthesizing functional products using a microbial cell factory. This achievement underscores the potential of metabolic engineering and synthetic biology in revolutionizing natural product biosynthesis.

**Supplementary Information:**

The online version contains supplementary material available at 10.1186/s12934-024-02448-4.

## Background

Alkaloids are naturally occurring compounds with a wide range of applications in agrochemicals and pharmaceuticals [1–3]. Chelerythrine, a benzophenanthridine alkaloid, is the primary functional component found in the Papaveraceae (*Chelidonium majus* L., *Sanguinaria canadensis* L., *Macleaya cordata* (Willd.) R.Br.) and Rutaceae (*Zanthoxylum asiaticum* L.) families. Due to it remarkable antibacterial, anti-inflammatory, and antioxidant activities, chelerythrine has been used as a growth-promoting feed additive in animal production [4–7]. With the worldwide ban on antibiotics growth promoters in food animals, chelerythrine has gained increasing demand as a green alternative [8]. Moreover, chelerythrine is used as a biopesticide to prevent and control crop epidemics [9]. However, due to its complex structure, achieving a total chemical synthesis of chelerythrine is challenging [10]. Currently, chelerythrine production mainly relies on direct plant extraction. However, its low yield (0.01–0.1% dry weight) and limited availability from natural sources restrict its industrial and clinical applications [11, 12].

Microbial cell factories, a promising alternative for synthesizing various plant alkaloids, can potentially reduce dependence on plant sources for sustainable production [13, 14]. DeLoache et al. successfully achieved the *de novo* biosynthesis of (*S*)-reticuline, a crucial intermediate for benzylisoquinoline alkaloids (BIAs), from glucose in *S. cerevisiae* with an initial titer of 80.60 µg/L [15]. Based on this, the production of (*S*)-reticuline was further enhanced to 4.60 g/L through metabolic pathway modification [16]. This achievement lays a foundation for the synthesis of downstream compounds, including chelerythrine and sanguinarine. Using a synthetic strain for (*S*)-reticuline as a platform with norlaudanosoline as the feeding substrate [17], Trenchard et al. demonstrated the synthetic pathway for sanguinarine in an optimized yeast strain, which required the heterologous expression of seven additional enzymes (Berberine bridge enzyme (BBE), cheilanthifoline synthase (CFS), stylopine synthase (STS), tetrahydroprotoberberine *cis*-*N*-methyltransferase (TNMT), (*S*)-*cis*-*N*-methylstylopine 14-hydroxylase (MSH), protopine 6-hydroxylase (P6H) and dihydrobenzophenanthridine oxidase (DBOX)) [13]. Nevertheless, to our knowledge, there is currently no research on the microbial biosynthesis of chelerythrine as a downstream product of (*S*)-reticuline.

Recently, Liu et al. demonstrated that the chelerythrine biosynthesis pathway in *Macleaya cordata*, which includes eight catalytic steps starting from (*S*)-reticuline. BBE catalyzes the first stereospecific oxidation and carbon-carbon bond formation step from (*S*)-reticuline to (*S*)-scoulerine. (*S*)-scoulerine-9-O-methyltransferase (SMT) and TNMT catalyze the methylation of (*S*)-scoulerine and (*S*)-canadine into (*S*)-tetrahydrocolumbamine and (*S*)-*N*-methylcanadine. (*S*)-tetrahydrocolumbamine, (*S*)-*N*-methylcanadine and allocryptopine are oxidized by P450 enzymes (*S*)-canadine synthase (TDC), MSH and P6H to form (*S*)-canadine, allocryptopine and 6-hydroxyallocryptopine, respectively. 6-hydroxyallocryptopine is spontaneously transformed to dihydrochelerythrine, which is oxidized to chelerythrine under catalysis of DBOX [18]. However, the low yield of chelerythrine and the accumulation of the intermediate allocryptopine in plants imply that the associated enzyme activities are also limited [18]. This emphasizes the formidable hurdles involved in optimizing the biosynthetic pathway. Addressing these challenges will require the construction of a synthetic pathway from (*S*)-reticuline to chelerythrine, combined with optimizing the catalytic efficiency of enzymes for each key catalytic step, regulating metabolic pathways, and controlling fermentation conditions.

In this study, the necessary enzymatic catalysts were selected from eight distinct plant species through meticulous analysis. The CRISPR-Cas9 genome editing technology was used to initially establish the biosynthetic pathway for chelerythrine by integrating the optimized enzymes into *S. cerevisiae* W303-1 A. Using a system-level regulatory strategy, the chelerythrine yield was significantly enhanced by overexpression rate-limiting gene, optimizing cofactors supply, and enhancing product transport. The chelerythrine titer increased to 12.61 mg/L in the best performing strain by pH-based fed-batch fermentation by optimizing the media and inoculum concentrations. Our study provides a reliable route for chelerythrine production, facilitating future research on bioactive alkaloids and their production.

## Methods

### Strains and media

*Escherichia coli* DH5α was used for gene cloning. Using *S. cerevisiae* W303-1 A (*MATα leu*2-3,112 *trp*1-1 *can*1-100 *ura*3-1 *ade*2-1 *his*3-11,15) as the background strain (Z0) for genetic manipulations and strain construction, strain Z0′ was generated by integrating a CPR cassette encoding cytochrome P450 reductase into strain Z0. Genes, including *AmBBE*, *EcBBE*, *McoBBE*, *tMcoBBE*, *PsBBE*, *AmSMT*, *CcSMT*, *PsSMT*, *TfSMT*, *CmTNMT*, *EcTNMT*, *GfTNMT*, *PsTNMT* were expressed in strain Z0, generating strains Z0(AmBBE), Z0(EcBBE), Z0(McoBBE), Z0(tMcoBBE), Z0(PsBBE), Z0(AmSMT), Z0(CcSMT), Z0(PsSMT), Z0(TfSMT), Z0(CmTNMT), Z0(EcTNMT), Z0(GfTNMT), Z0(PsTNMT). Genes, including *EcP6H*, *t4EcP6H*, *t27EcP6H*, *McoP6H*, *PsP6H*, were expressed in strain Z0’, generating strains Z0‘(EcP6H), Z0‘(t4EcP6H), Z0‘(t27EcP6H), Z0‘(McoP6H), Z0‘(PsP6H). *t4EcP6H*, and *t27EcP6H* encode mutants with 4- and 27-residue deletion of EcP6H, respectively. Strains Z1–Z22 were obtained through genome integration of genes, while all other strains were obtained through plasmid importation unless otherwise specified. All the strains and plasmids used in this study are listed in Additional file 1: Table S1.

*E. coli* strains were selected from Luria-Bertani (LB) agar plates and grown in liquid LB medium containing 50 mg/L ampicillin. Yeast cells were cultivated in YPD medium containing 20 g/L glucose, 20 g/L peptone, and 10 g/L yeast extract. Strains containing URA3- or/and TRP1-based plasmids/cassettes were selected from synthetic complete media (SC1/SC2/SC medium) without uracil or/and tryptophan (SC-URA/SC-TRP/SC-TRP-URA, 20 g/L glucose).

### Genetic engineering

pRS416 was used as the empty vector to construct the expression vector. Promoters and terminators were amplified from the Z0 genome. Gene overexpression cassettes containing a promoter, target gene, terminator, and block of the next cassette (homologous sequences between contiguous expression cassettes) for homologous recombination were constructed using overlapping extension PCR. The overexpression plasmid was constructed by digestion and ligation. Guide RNA (gRNA) plasmids targeting various genome sites were designed using CRISPRdirect (http://crispr.dbcls.jp) [19] and constructed using PCR and in-fusion cloning. Clones were verified using colony PCR and sequencing. The gRNA targeting sequences are listed in Additional file 1: Table S2. All codon-optimized heterologous genes and primers are listed in Additional file 1: Table S3 and S4, respectively.

All expression cassettes were integrated into their designated chromosomal sites to provide stable and high-level expression of heterologous genes. Genome editing was performed using p414-TEF1p-Cas9-CYC1t (ID: 43,802), which contains the Cas9 cassette, and the gRNA-expressing plasmid p426-SNR52p-gRNA.CAN1.Y-SUP4t (ID: 43,803). The CRISPR-Cas9 method was used to co-transform the gel-purified overexpression cassettes and a gRNA plasmid into the parental strain for cassette knock-in, as previously reported [19]. After verification using colony PCR and sequencing, the clones were plated on an SC2 solid medium containing 5-fluoroorotic acid (5-FOA) to remove the gRNA vectors and recycle the URA3 marker.

### Yeast cultivation assays for the production of chelerythrine and its precursors

Cultures were grown at 30 °C and a shaking speed of 260 rpm for 12–18 h. They were then back-diluted with 5 × SC or 5 × YPD medium and grown in a 10 mL sterile tube with an initial optical density at 600 nm (OD_600_) of 0.2 for 36 h. After centrifuging each culture at 500 × *g* for 4 min, the supernatant was discarded to collect the cells. During chelerythrine synthesis, the cells pellets were added in 1 mL of SC or YPD media containing 100 µM of the substrate ((*S*)-reticuline or (*S*)-canadine) and cultured for 10 days at 28 °C, unless otherwise specified. Although different substrates were used to produce other intermediates, the culture method remained the same. All measurements were performed in triplicate.

Seed culture was produced by growing the engineered strain Z22 from a freshly streaked plate in SC media at 30 °C for 36 h and then back-diluting it into 5 × YPD media. The culture was then grown at an initial OD_600_ of 0.2 for 48 h. Fermentation was initiated by introducing the seed culture into a 0.5 L fermentation vessel (T&J-Quickflow 16-channel, Shanghai, China) containing 0.15 L of YPD, with a final OD_600_ of 40. The fermentation was performed at 28 °C with initial stirring at 300 rpm. The airflow was maintained at an initial rate of 1 vessel volume per minute (vvm) using a compressor (GA-81Y 220 V, Shanghai, China). The dissolved oxygen (DO) level was maintained above 40% saturation via an automated cascade by increasing the stirring rate to 1200 rpm and airflow to 2 vvm. Further, 30% NH_4_OH was automatically added to maintain the pH at 4.5. Feeding was automatically controlled by the pH-based feedback feeding method. Feeding was initiated at a pH ≥ 4.5, and a base was added when it fell below this level. The feeding solution consisted of 500 g/L glucose, 9 g/L KH_2_PO_4_, 2.5 g/L MgSO_4_, 3.5 g/L K_2_SO_4_, 0.28 g/L Na_2_SO_4_, 10 mL/L trace metal solution, and 12 mL/L vitamin solution was used to sustain cell growth and activity [20]. Fermentation lasted for 8 days, and samples were taken at intervals of 24 h.

### Metabolite extraction and quantification

Extraction of intermediate metabolites: Metabolites were extracted from the culture broth containing cells and growth medium and then analyzed using liquid chromatograph-mass spectrometry (LC-MS) or LC-tandem mass spectrometry (LC-MS/MS) on an Agilent 6300 system. Specifically, after centrifuging the cell culture at 1,300 × *g* for 30 min, the precipitate was supplemented with an equal volume of ethanol. The precipitated mixture was then homogenized with acid-washed glass beads (425–600 μm) by a high-throughput tissue grinder (60 Hz, 30 min). The suspension was centrifuged to obtain the supernatant. The cell pellet and acid-washed glass beads were resuspended, vortexed and centrifuged to obtain the supernatant. These supernatants were mixed, centrifuged, and subjected to LC-MS for product detection.

Quantification of intermediate metabolites: Samples were separated using quadrupole-time of flight mass spectrometry equipped with the electrospray positive ion mode on an Agilent EclipsePlus C18 column (2.1 × 50 mm, 1.8 μm) using 0.1% formic acid in water (solvent A) and 0.1% formic acid in acetonitrile (solvent B). The quantification of metabolites was carried out using a constant flow rate of 0.4 mL/min for a specific duration, divided into four segments of 0–0.1 min (10% B), 0.1–5 min (10–45% B), 5–5.5 min (45–90% B), and 5.5–7.01 min (90–10% B), followed by a 3-minute equilibration using a 10% solvent B. The samples were quantified by calculating the integra ted peak area of the extracted ion chromatogram peaks. Due to the absence of a standard for (*S*)-*N*-methylcanadine, its concentration was calculated based on the amount of substrate consumed. For MS/MS analysis, spectra were acquired with a 0.3 s scan time and the selected ion was subjected to a collision energy ramp of 2–40 eV. The standard chelerythrine and metabolism-related intermediates were purchased from Chengdu purechem-standard Co., LTD (Chengdu, China).

Cellular NADPH/NADP^+^ quantification: Cellular NADPH/NADP^+^ was quantified using the CheKine NADP^+^/NADPH assay Kit (catalog no. WST-8; Abbkine). After 36 h of culture, the cells were centrifuged and resuspended in ice-cold PBS buffer to an OD_600_ of 10. The samples were extracted with the NADPH/NADP^+^ extraction buffer and all subsequent steps were performed according to the manufacturer’s instructions.

### Statistics and reproducibility

All experiments were performed at least in triplicate. The data was expressed as means ± standard errors. All data analysis was performed by Excel, OriginPro, or GraphPad Prim 9.

## Results

### Reconstruction of catalytic modules for the heterologous biosynthesis of chelerythrine

**Fig. 1:**
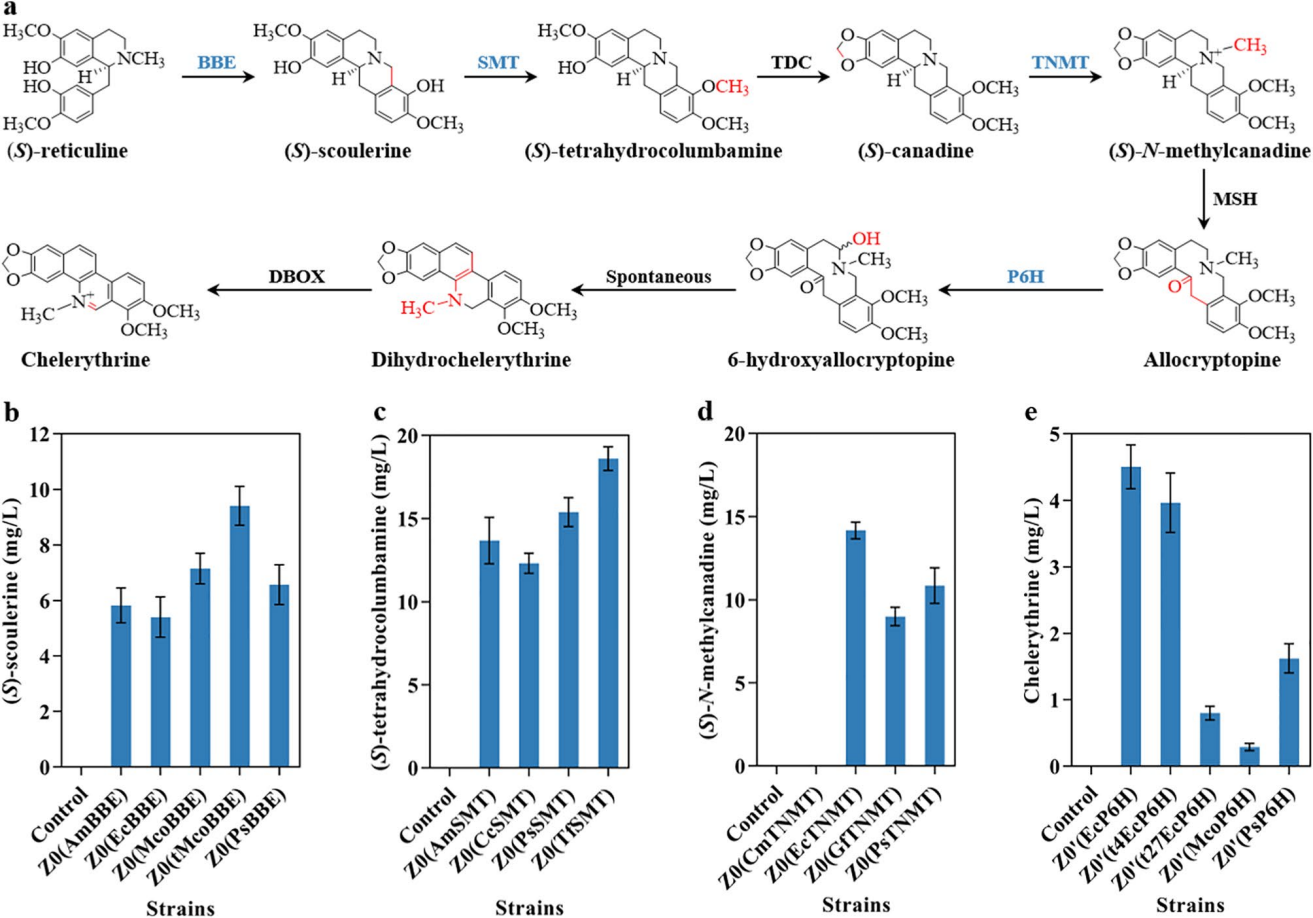
The biosynthetic pathway of chelerythrine and screening of candidate enzymes. **a** Biosynthetic pathway of chelerythrine starting from (*S*)-reticuline. **b** Screening BBE for (*S*)-scoulerine production in yeast cells using 100 µM (*S*)-reticuline as the substrate. **c** Screening SMT for (*S*)-tetrahydrocolumbamine production in yeast cells using 100 µM (*S*)-scoulerine as the substrate. **d** Screening TNMT for (*S*)-*N*-methylcanadine production in yeast cells using 100 µM (*S*)-canadine as the substrate. **b**-**d** Background strain Z0 with an empty vector pRS416 was used as the control. **e** Chelerythrine detection for P6H selection using 100 µM allocryptopine as the substrate. Strain Z0′ with an empty vector pRS416 was used as the control

To establish the biosynthetic pathway for chelerythrine from (*S*)-reticuline, heterologous genes, including *McoBBE*, *AmSMT*, *AmTDC*, *PsTNMT*, *PsMSH*, *PsP6H*, *McoDBOX*, and *PsCPR* from Papaveraceae plants involved in the synthesis of chelerythrine and its precursor, were introduced into strain Z0 (Fig. 1a). The fusion of target genes with green fluorescent protein validated expression of each gene in Z0 (Additional file 1: Fig. S1). After 10 days of culture at 28°C in a 10 mL tube, chelerythrine accumulation could not be detected by LC-MS because of the enzyme imbalance in the metabolic pathway, indicating that the enzymatic activity involved in catalyzing the pathway might be deficient.

BBE, which catalyzes the initial stereospecific oxidation and addition reaction step from (*S*)-reticuline to (*S*)-scoulerine, is the rate-limiting enzyme in the sanguinarine biosynthesis pathway [21, 22]. To select highly efficient enzymes for (*S*)-scoulerine synthesis in Z0, four BBEs from Papaveraceae plants, namely *Papaver somniferum* PsBBE [17], *Argemone mexicana* AmBBE [23], *Eschscholzia californica* EcBBE [24], and *M. cordata* McoBBE [18], were compared on the basis of catalytic efficiency for producing (*S*)-scoulerine in yeast. LC-MS analysis of (*S*)-scoulerine production after providing 100 µM (*S*)-reticuline extracellularly indicated that strain Z0(McoBBE) produced a higher concentration of (*S*)-scoulerine with a conversion efficiency of 21.92% from (*S*)-reticuline (Fig. 1b). Considering that BBE is localized in the vacuole [25], we designed a truncated mutant, tMcoBBE, with a 24-residue deletion based on the transit peptide prediction by SignalP-5.0 (Additional file 1: Fig. S2). This mutant was expressed in the cytosol and exhibited a 31.42% increase in the production of chelerythrine compared to that of McoBBE (Fig. 1b, Additional file 1: Fig. S3).

SMT catalyzes the transfer of the *S*-methyl group from *S*-adenosyl-L-methionine to the 9-hydroxyl group of (*S*)-scoulerine, leading to the formation of (*S*)-tetrahydrocolumbamine [26]. Four SMTs, including *A. mexicana* AmSMT, *Coptis chinensis* CcSMT, *P. somniferum* PsSMT, and *Thalictrum flavum* TfSMT mutant (N191D, F205S) [27], were selected and heterologously expressed in strain Z0, respectively. Among these candidates, the TfSMT mutant exhibited exceptional performance, producing 18.61 mg/L of (*S*)-tetrahydrocolumbamine when 100 µM (*S*)-scoulerine was used as the substrate (Fig. 1c). To optimize the key enzymes involved in this process, TNMT and P6H were screened from different plant sources based on substrate similarity. The results showed that EcTNMT and EcP6H from *E. californica* efficiently increased chelerythrine production (Fig. 1d and e).

**Fig. 2:**
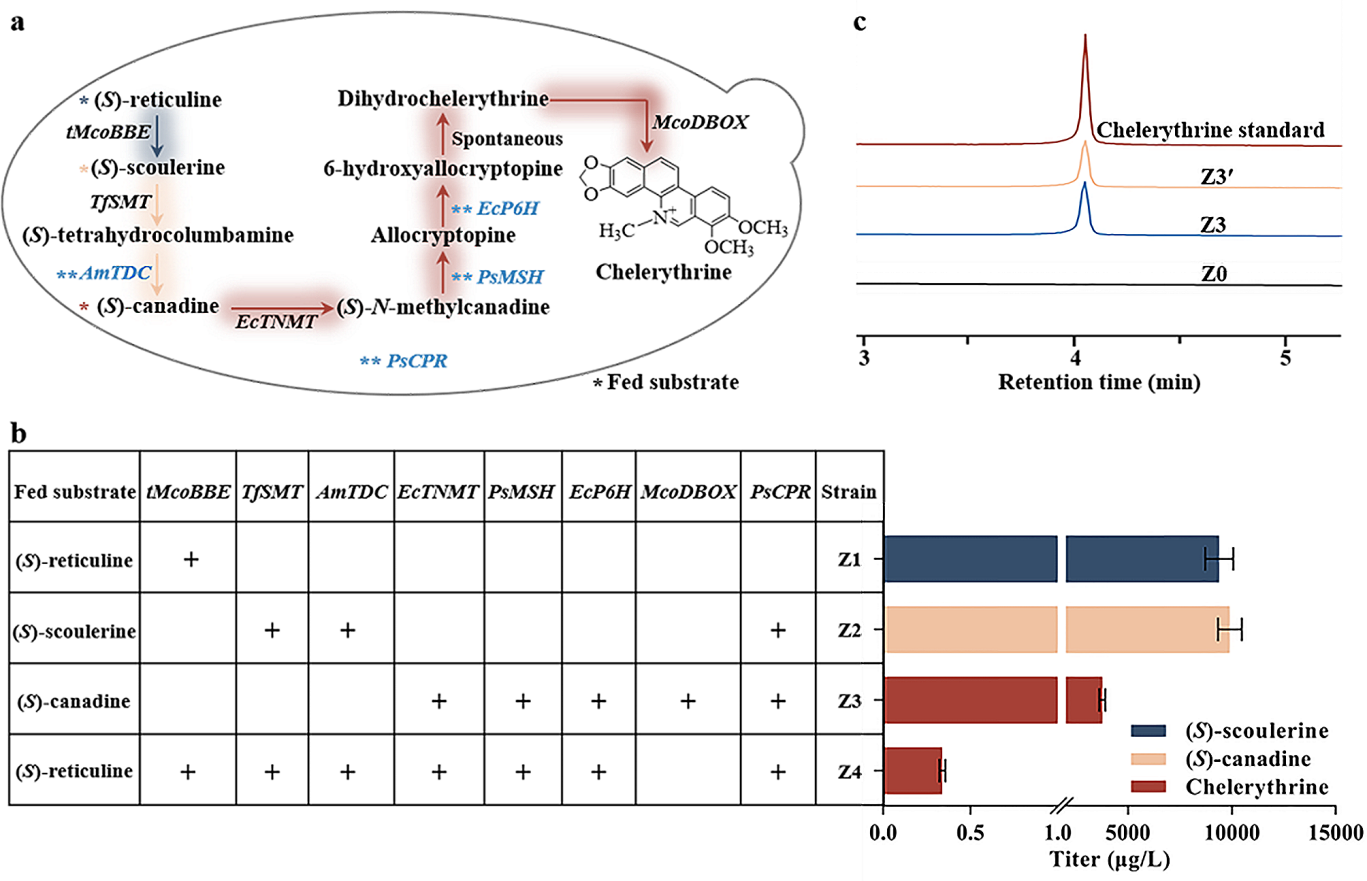
Reconstructed biosynthetic pathway of chelerythrine. **a** Biosynthetic pathway from (*S*)-reticuline to chelerythrine in *S. cerevisiae*. **b** Modular assembly of chelerythrine biosynthesis. **c** Extraction ion chromatograms of chelerythrine from strains Z0, Z3, Z3′, and its standard. The recombinant strains were cultured for 10 days in SC medium containing 100 µM substrate, and the resulting metabolites were analyzed by LC-MS

Three functional modules (modules A, B, and C), corresponding to carbon-carbon formation, preliminary methylation/oxidation, and multiple methylation/oxidation reactions, respectively, were used to design the synthetic pathway for chelerythrine from (*S*)-reticuline (Fig. 2a). For the carbon-carbon bond formation module, *tMcoBBE* was inserted into the chromosome of strain Z0 to generate the engineered yeast Z1 for (*S*)-scoulerine biosynthesis from (*S*)-reticuline at a conversion rate of 28.62% (Fig. 2b). Module B includes SMT and TDC, the two enzymes essential for transforming (*S*)-scoulerine to (*S*)-canadine. Due to its high catalytic and specific performance [28], *A. mexicana* AmTDC was used directly for the construction of the chelerythrine pathway. Considering that cytochrome P450 monooxygenases, such as TDC, require reducing equivalents to enhance their activity, which are provided by redox partner proteins containing cytochrome P450 reductases (CPRs) to transfer electrons [29], the *TfSMT* mutant, *AmTDC*, and *P. somniferum PsCPR* gene expression cassettes were integrated into Z0, generating strain Z2. When 100 µM (*S*)-scoulerine was added to the SC medium, strain Z2 synthesized (*S*)-canadine at a titer of 9.91 mg/L (Fig. 2b). Module C for synthesizing chelerythrine from (*S*)-canadine involves four enzymes: TNMT, MSH, P6H, and DBOX. Therefore, codon-optimized expression cassettes encoding EcTNMT, PsMSH [30], EcP6H, McoDBOX [18], and PsCPR were integrated into strain Z0, generating strain Z3. Using 100 µM (*S*)-canadine as the substrate, this strain was able to produce 3.76 mg/L chelerythrine (Fig. 2b). Chelerythrine synthesis was also observed in engineered yeast Z3’ even without integrating *McoDBOX* into strain Z0 (Fig. 2c), indicating the presence of an endogenous enzyme or enzymes that can catalyze the final step of chelerythrine synthesis. Given its higher maximum specific growth rate (strain Z3’, 0.35 h^− 1^ and strain Z3, 0.30 h^− 1^, respectively) and comparable chelerythrine synthesis levels to strain Z3 (Additional file 1: Fig. S4), strain Z3’ was selected as the platform strain for constructing the chelerythrine synthesis pathway. To construct a strain capable of producing chelerythrine from (*S*)-reticuline, the genome of the strain Z3’ was modified by integrating gene expression cassettes encoding tMcoBBE, TfSMT, and AmTDC, generating strain Z4. LC-MS and LC-MS/MS analysis confirmed that Z4 could successfully produce chelerythrine at a titer of 0.34 µg/L, which matched the chelerythrine standard (Fig. 2b, Additional file 1: Fig. S5).

### Fine-tuning of the chelerythrine biosynthesis pathway via integrating multiple-copy rate-limiting genes

To enhance the metabolic flux, the chelerythrine biosynthesis pathway was systematically optimized by examining the impact of multiple copies of each enzyme, especially P450s, on chelerythrine synthesis in Z4, selecting and integrating multiple-copy rate-limiting genes.

**Fig. 3:**
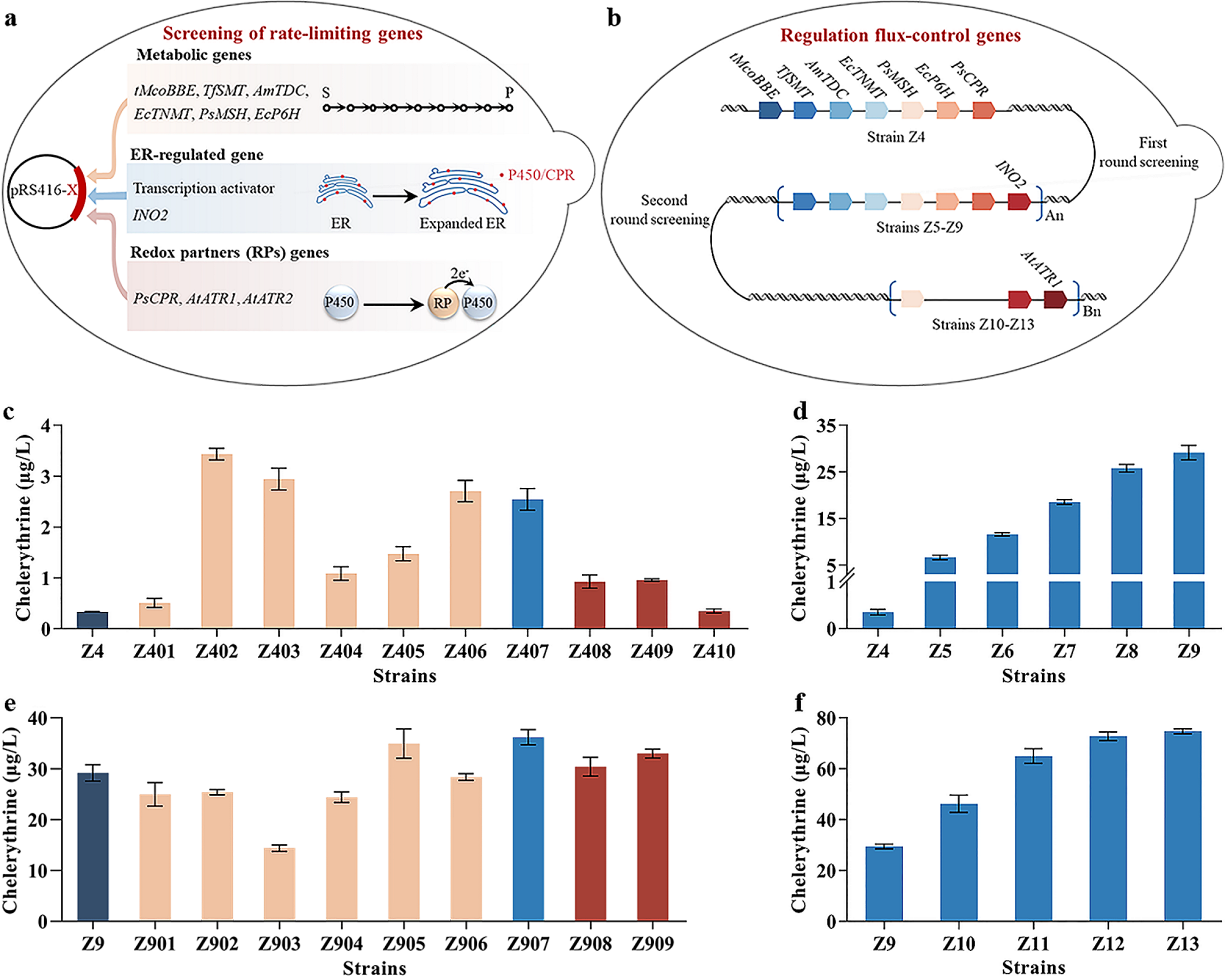
Multifaceted regulation of the rate-limiting P450 steps and tuning the expression of flux-control genes for efficient chelerythrine production. **a** Schematic diagram of the screening process for rate-limiting genes in strain Z4 or Z9, including those in the pathway and those beneficial for enhancing P450s performance to enhance chelerythrine titer. S and P denote substrate (*S*)-reticuline and product chelerythrine, respectively. **b** Schematic diagram for regulating the expression of flux-control genes to improve chelerythrine synthesis. **c** Screening of rate-limiting genes for efficient chelerythrine production in strain Z4. **d** Improved chelerythrine titer owing to the integrated multigene cassettes A in strain Z4. **e** Screening of rate-limiting genes for efficient chelerythrine production in strain Z9. **f** Improved chelerythrine titer owing to the integrated multigene cassettes B in strain Z9. The recombinant strains were cultured for 10 days in SC medium containing 100 µM (*S*)-reticuline, and the resulting metabolites were analyzed by LC-MS

First, individual genes *tMcoBBE*, *TfSMT*, *AmTDC*, *EcTNMT*, *PsMSH*, and *EcP6H* were overexpressed in strain Z4 using the plasmid pRS416 (Fig. 3a), resulting in strains Z401–Z406, respectively. It was found that overexpression of all metabolic genes improved chelerythrine yield to varying degrees (Fig. 3c), indicating that amplified gene copy numbers in the pathway enhanced the synthesis of chelerythrine.

The expression of multiple P450s can be challenging and often leads to metabolic bottlenecks in heterologous biosynthesis pathways, as reported in previous studies [13, 31, 32]. In our study, the high sensitivity for overexpression of P450 genes (*TDC*, *MSH*, and *P6H*, individually) in strains Z403, Z405, and Z406, resulting in a 5- to 10-fold increase in chelerythrine production (Fig. 3c), suggests that P450 activity was limited. To address this issue, the P450s were systematically optimized in terms of their location size, expression levels, and catalytic efficiency. Considering that heterologously expressed plant-derived P450s and CPRs are located in the endoplasmic reticulum (ER) [28, 33], the size of the ER is closely related to its protein synthesis and folding capacity [34, 35]. To enhance the attachment sites for P450 overexpression and activity enhancement, we amplified a key transcription factor, *INO2*, which is involved in terpene synthesis [33], to expand the ER’s size (Fig. 3a). The resulting strain Z407 had a significantly higher chelerythrine yield of 2.54 µg/L, demonstrating a 7.47-fold improvement over the parental strain Z4 (Fig. 3c). Moreover, alternative plant CPRs, such as PsCPR, *Arabidopsis thaliana* AtATR1, and AtATR2 [36], were expressed to enhance P450 activity in chelerythrine synthesis (Fig. 3a). Our findings revealed that overexpression of these specific genes, excluding *AtATR2*, significantly enhanced chelerythrine production in both Z408 and Z409, which expressed *PsCPR* and *AtATR1* in strain Z4, with titers of 0.93 and 0.95 µg/L, respectively, representing a 173.53% and 179.41% increase compared to strain Z4 (Fig. 3c). This indicates that overexpression of CPR can significantly increase P450 activity in the chelerythrine biosynthesis pathway.

To further boost chelerythrine synthesis, multigene cassettes A, comprising rate-limiting genes *TfSMT*, *AmTDC*, *EcTNMT*, *PsMSH*, *EcP6H*, *PsCPR*, and *INO2*, was integrated into the genome of strain Z4. Multiple copies of these genes were added to generate strains Z5-Z9 (Fig. 3b). These strains showed a significant improvement in chelerythrine titer, with strain Z9 producing 29.15 µg/L, which was approximately 83 times higher than that of strain Z4 (Fig. 3d).

A second screening for rate-limiting genes was performed in strain Z9 to further optimize the metabolic pathway (Fig. 3a). Overexpression of the individual genes *tMcoBBE*, *TfSMT*, *AmTDC*, *EcTNMT, PsMSH*, *EcP6H*, *INO2*, *PsCPR*, and *AtATR1* in strain Z9 resulted in strains Z901 to Z909, respectively. However, the increase in chelerythrine yield was significant only in the strains overexpressing of *MSH*, *ATR1*, and *INO2* (Fig. 3e). Thus, multigene cassettes B, including the rate-limiting genes *MSH*, *ATR1*, and *INO2*, was integrated into strain Z9, generating various copies of engineered strains Z10–Z13 (Fig. 3b). Through two rounds of iterative integration of multiple-copy rate-limiting genes, strain Z13 was obtained, which produced the highest chelerythrine yield, at 74.74 µg/L (Fig. 3f).

### Engineering heme supply to increase chelerythrine production

**Fig. 4:**
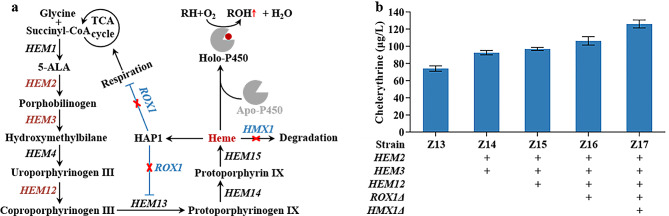
Regulation of the heme supply for efficient chelerythrine production by enhancing the efficiency of P450s. **a** Schematic illustration of the genetic modifications performed to relieve potential metabolic limitations, regarding heme metabolism, to improve the performance of P450s. *HEM1* (encoding 5-aminolevulinic acid synthase), *HEM2*, (encoding porphobilinogen synthase), *HEM3* (encoding porphobilinogen deaminase), *HEM4* (encoding uroporphyrinogen III synthase), *HEM12* (encoding uroporphyrinogen III decarboxylase), *HEM13* (encoding coproporphyrinogen III oxidase), *HEM14* (encoding protoporphyrinogen IX oxidase), *HEM15* (encoding ferrochelatase), *HMX1* (encoding heme oxygenase). *ROX1*, a heme-dependent repressor of the hypoxic gene. HAP1, a transcriptional activator. **b** Chelerythrine production in engineered yeast strains derived from enhancing intracellular heme supply by strengthening synthesis and reducing heme degradation. The recombinant strains were cultured for 10 days in SC medium containing 100 µM (*S*)-reticuline, and the resulting metabolites were analyzed by LC-MS

Sufficient amounts of heme, a cofactor, are required to assemble active P450 holoenzymes. The depletion of the intracellular pool of heme triggers cellular stress, which might hamper enzymatic activity [37]. To address this challenge, three strategies were employed to engineer heme biosynthesis: strengthening rate-limiting enzymes, eliminating feedback inhibition, and reducing heme degradation (Fig. 4a).

Co-overexpression of the rate-limiting enzymes HEM2 and HEM3 [38] improved the chelerythrine production in strain Z14 (92.54 µg/L) by 24.90% compared to the parental strain Z13 (Fig. 4b). Further improvement was achieved in Z15 through combined overexpression of HEM12, a key enzyme in heme biosynthesis. Deletion of *ROX1*, which restrains HEM13 activity and mitochondrial respiration [31, 38], led to a rise in chelerythrine production, with a yield of 106.36 µg/L in strain Z16 (Fig. 4b).

Compared with strain Z16, deletion of *HMX1* gene encoding heme oxygenase responsible for heme degradation [39] further improved chelerythrine production by 18.52% in the Z17 strain (126.06 µg/L) (Fig. 4b). A previous study showed that feeding 5-aminolevulinic acid (5-ALA), the immediate precursor of heme biosynthesis, as the substrate markedly increased the intracellular heme level in yeast [37]. The effect of external supplementation with 5-ALA on chelerythrine production was also investigated, but no significant increase was observed (data not shown), indicating that 5-ALA was not a limiting precursor in this case.

### Engineering NADPH supply increasing the titer of chelerythrine

**Fig. 5:**
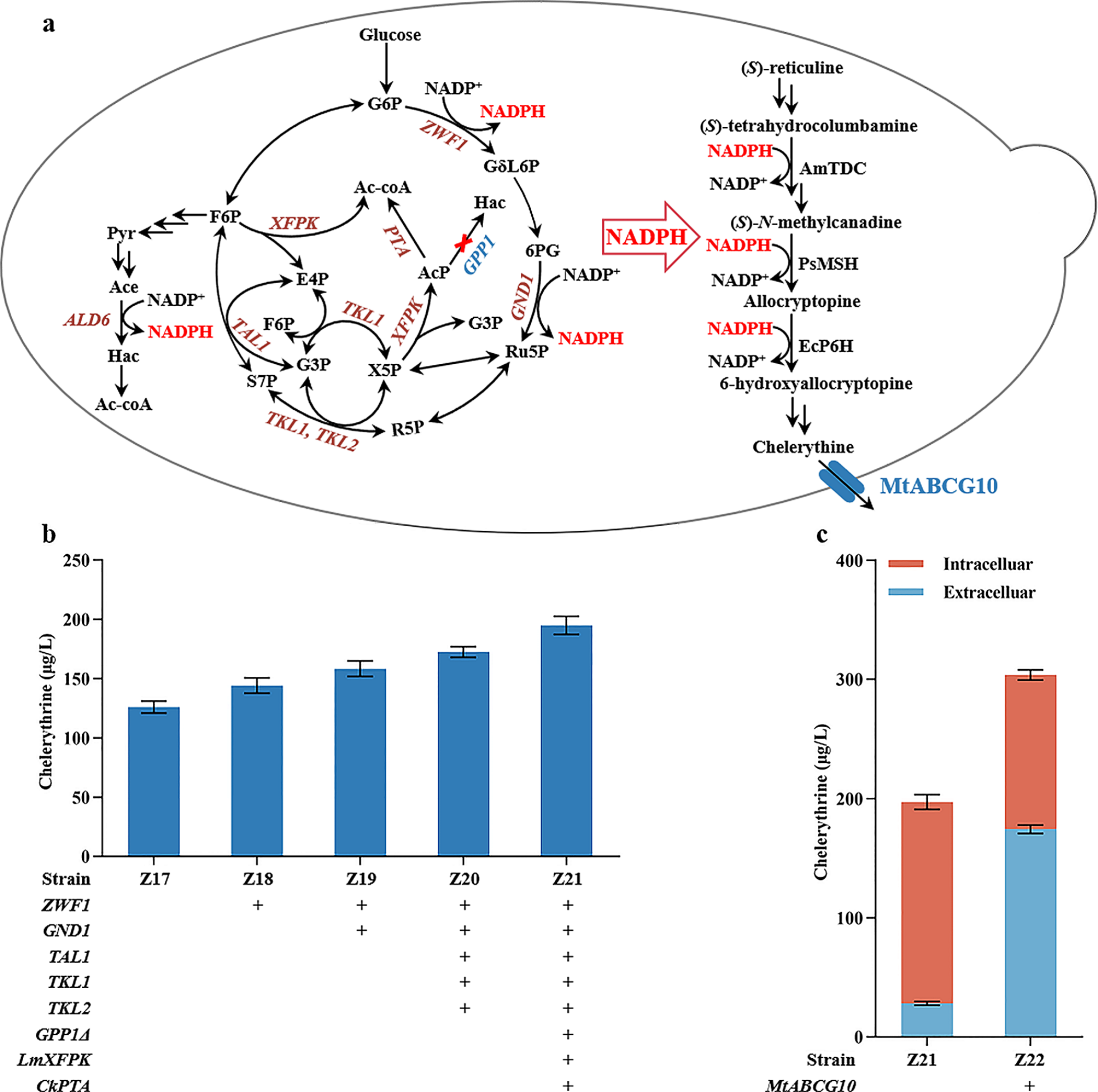
NADPH regeneration and product extracellular transport regulation for optimal chelerythrine biosynthesis. **a** Schematic illustration of NADPH regeneration for facilitating chelerythrine biosynthesis and alleviation of chelerythrine transport limitations through heterologous transporter expression in engineered yeast. G6P, Glucose-6-phosphate; GδL6P, 6-phosphogluconolactone; 6PG, 6-phosphogluconate; Ru5p, Ribulose 5-phosphate; R5P, Ribose 5-phosphate; X5P, Xylose 5-phosphate; S7P, Sedoheptulose 7-phosphate; G3P, Glyceraldehyde-3-phosphate; E4P, Erythrose-4-phosphate; F6P, Fructose 6-phosphate; Pyr, Pyruvicacid; AcP, Acetyl-phosphate; Ac-CoA, Acetyl-CoA; Ace, Acetaldehyde; Hac, Acetate. Heterologous expression of the blue-labeled transporter MtABCG10. **b** Chelerythrine production by engineering NADPH supply. **c** Intracellular and extracellular distribution of chelerythrine in engineered strain Z21 and Z22 with expression of heterologous transporter. The recombinant strains were cultured for 10 days in SC medium containing 100 µM (*S*)-reticuline, and the resulting metabolites were analyzed by LC-MS

Given that NADPH is a critical electron transfer cofactor in several catalytic steps of the chelerythrine biosynthesis pathway (Fig. 5a), NADPH deficiency might reduce P450 activity due to inefficient electron transfer. To increase chelerythrine yields, the *ALD6* gene, which encodes cytosolic NADP^+^-dependent acetaldehyde dehydrogenase [40], was introduced into strain Z17 to enhance NADPH production. However, the chelerythrine titer did not increase significantly in this strain and the NADPH/NADP^+^ ratio remained low (Additional file 1: Fig. S6, Fig. S7), indicating that intracellular NADPH was still inadequate and required enhanced regeneration. The pentose phosphate pathway (PPP) has been identified as the primary source of NADPH (Fig. 5a). Integrating the key gene *ZWF1* in PPP, encoding glucose-6-phosphate dehydrogenase, into strain Z17 resulted in strain Z18, which produced 144.22 µg/L of chelerythrine, representing an increase by 14.39% (Fig. 5b). Nevertheless, despite this improvement in the chelerythrine titer, the NADPH/NADP^+^ ratio in Z18 remained insufficient. To further increase NADPH supply, *GND1*, which encodes 6-phosphogluconate dehydrogenase that catalyzes the second oxidative reduction of NADP^+^ to NADPH in PPP, was introduced into strain Z18, generating strain Z19. Compared to strain Z18, the chelerythrine titer increased by 9.83% to 158.40 µg/L (Fig. 5b).

To enhance the PPP flux, genes *TKL1*, *TKL2*, and *TAL1*, which encode two transketolase and one transaldolase, respectively, were overexpressed to generate strain Z20, which produced chelerythrine at a titer of 172.51 µg/L. This is an 8.91% increase over that in strain Z19 (Fig. 5b). In addition, by combinatorial expression *Leuconostoc mesenteroides LmXFPK*, encoding phosphoketolase, and *Clostridium kluyveri CkPTA*, encoding phosphotransacetylase, with the deletion of *GPP1*, encoding GAP phosphatase, the resulting strain Z21 produced 195.03 µg/L chelerythrine (Fig. 5b), accounting for a 54.69% increase compared with that of strain Z17. These findings showed that *ZWF1*, *GND1*, *TAL1*, *TKL1*, *TKL2*, *XFPK*, and *PTA* involved in the PPP pathway could increase the NADPH/NADP^+^ ration and thereby increasing chelerythrine production. Notably, previous metabolic flux analysis suggested that downstream reversible steps were rate-limiting steps during glucose-limited conditions [41, 42], underscoring the importance of addressing the NADPH issue in promoting chelerythrine production during the later stages of synthesis.

### Engineering product trafficking to enhance chelerythrine secretion

Regulation of the synthesis pathway greatly enhanced chelerythrine production in the engineered yeast. However, chelerythrine predominantly accumulated intracellularly in strain Z21 and required extracellular transport to promote further synthesis. Several plant-derived transporters have been characterized for alkaloid transport to address the accumulation of some alkaloids in plants [43]. Four transporters, including *M. cordata* McoABC [18], *Medicago truncatula* MtABCG10 [44], *Catharanthus roseus* CrTPT2 [45], and *Coptis japonica* CjABCB2 [46], were integrated into strain Z21, respectively, to achieve extracellular transport of chelerythrine. Fortunately, the modified strain Z22, which integrated the gene *MtABCG10*, was highly effective in reducing intracellular accumulation, thereby enhancing the extracellular accumulation of chelerythrine, with the total titer increasing by 54.06% to 303.74 µg/L (Fig. 5c). The intracellular to extracellular ratio of chelerythrine was altered from 6:1 (169.00 to 28.16 µg/L, strain Z21) to 0.74:1 (129.43 to 174.31 µg/L, strain Z22), whereas the accumulation of chelerythrine in other recombinant strains remained unchanged (Fig. 5c, Additional file 1: Fig. S8). The ratio of extracellular to intracellular precursors, namely allocryptopine and dihydrochelerythrine, in strain Z22 increased significantly compared to that in strain Z21 (Additional file 1: Fig. S9).

### Fermentation process of the best-performing recombinant strain

**Fig. 6:**
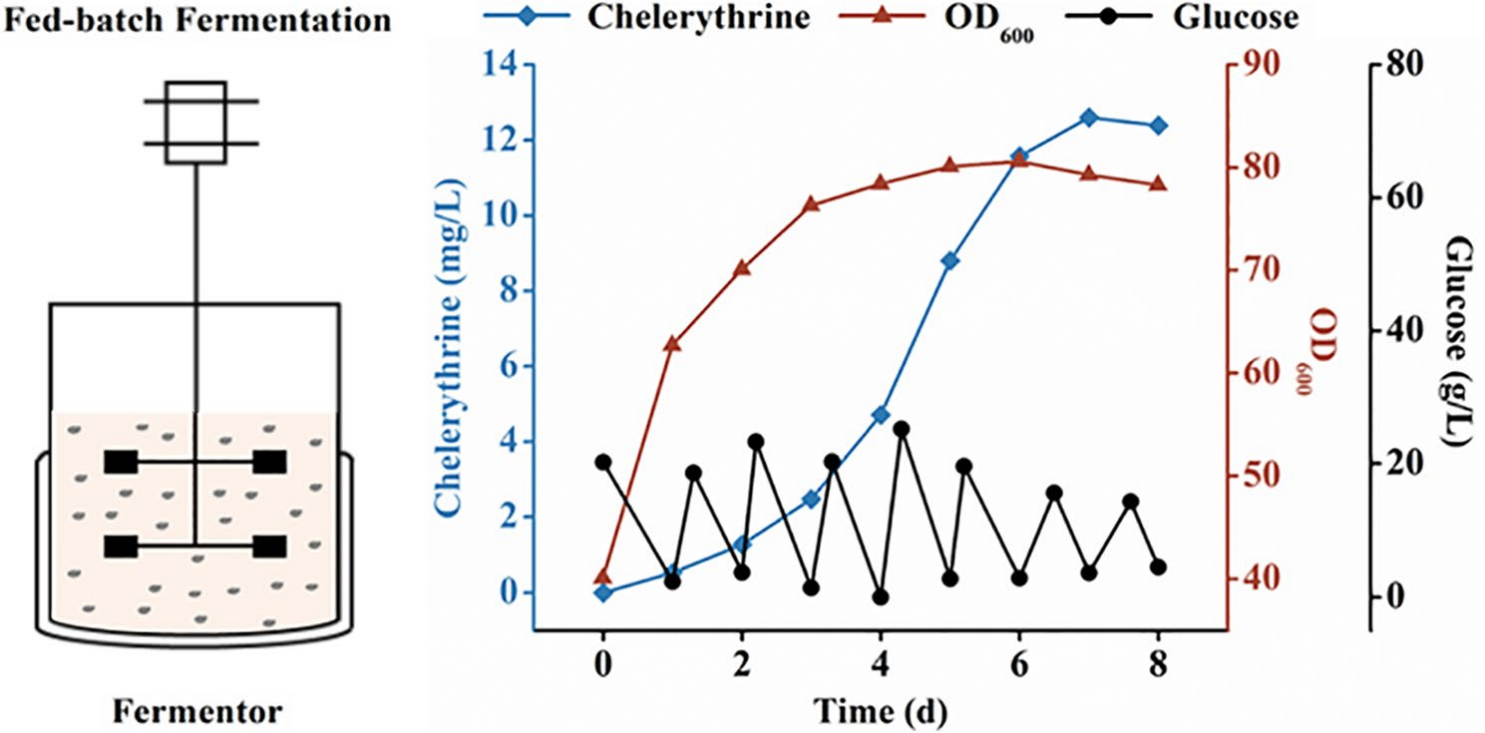
Fed-batch fermentation for chelerythrine production. The pH-based fed-batch fermentation was conducted using strain Z22 with an initial inoculum concentration of OD_600_ of 40. Yeast fermented in YPD medium was fed with 50 µM of (*S*)-reticuline at 48-hour intervals. Fermentation was maintained for 8 days with a total substrate (*S*)-reticuline concentration of 200 µM

Experimental fermentation conditions, including the impact of varying initial inoculum concentrations and culture media on the accumulation of the target compound, were optimized to improve the chelerythrine titer. As the inoculum level increased, there was a corresponding increase in chelerythrine accumulation per unit volume (Additional file 1: Fig. S10). When the OD_600_ of the initial inoculum concentration was 40, the production of chelerythrine in strain Z22 reached 1214.47 µg/L and maintained this level after that (Additional file 1: Fig. S10). Feeding experiments carried out in the YPD medium with strain Z22 demonstrated that the chelerythrine yield increased by 78.01% to 2161.82 µg/L when compared with the SC medium in 10 days. Using an optimized culture medium and inoculum concentration, the chelerythrine titer was raised to 12.61 mg/L in the best-performing strain Z22 through pH-based fed-batch fermentation in a 0.5 L bioreactor (Fig. 6).

## Discussion

Developing a practical biosynthesis pathway for alkaloids using yeast presents a considerable challenge because of the limited endurance of the strictly regulated internal network of microbial cells to exogenous polygenic systems [47]. To increase the efficiency of the reconstructed pathway in yeast, we performed comprehensive strategies, including multi-enzyme screening and optimization, metabolic pathway regulation, cofactor supply, and product transport.

The catalytic efficiency of enzymes significantly impacts product synthesis [15–17]. Screening enzymes with high catalytic efficiency based on their homology for pathway construction is an effective strategy. Using this strategy, Liu et al. identified the key enzymes involved in the biosynthesis of bioactive isoflavonoids [31]. We applied an enzymatic screening strategy based on substrate structural similarity and enzyme homologs to characterize relevant enzymes from the Ranunculales family and employ targeted cyclization, multiple methylation and hydroxylation synthesis systems. Four enzymes (tMcoBBE, TfSMT, EcTNMT, EcP6H) with higher catalytic efficiency in the synthesis pathway of chelerythrine were efficiently achieved (Fig. 1b-e), completing the initial construction of the artificial biosynthesis pathway. The first generation of engineered yeast yielded 0.34 µg/L chelerythrine, laying the foundation for subsequent pathway improvements. This approach rapidly narrows the range of candidate enzymes to produce highly efficient target enzymes. However, there may be an imbalance in metabolic pathways, resulting in extremely low efficiency in chelerythrine synthesis.

Overexpression of rate-limiting enzymes is often employed to regulate metabolic pathways and enhance engineered strain biosynthesis [47]. Optimizing the selection of promoters and overexpressing genes is commonly used to increase the gene copy number. Trenchard et al. enhanced the biosynthesis of cheilanthifoline in yeast by selecting an optimal promoter for regulating the expression of rate-limiting P450 [13]. Pyne et al. enhanced the biosynthesis of (*S*)-reticuline by overexpressing rate-limiting norcoclaurine synthase [16]. To increase the titer of synthetic chelerythrine, we implemented a rate-limiting gene screening and iterative gene assembly strategy to screen and increase the expression of rate-limiting enzymes. Conceptually, each gene was overexpressed individually, and its perturbation on product synthesis was observed to determine the sensitivity of the yeast system to enzyme requirements (Fig. 3c and e). Based on this, gene copies were customized in the yeast genome to increase the yield of the target product. The results indicated that increasing the copies of pathway genes (such as *TfSMT*, *AmTDC*, *EcTNMT*, *PsMSH*, and *EcP6H*) and P450 activity-related genes (*INO2*, *PsCPR*) can significantly improve chelerythrine synthesis, with a yield of up to 83 times. Using repetitive rounds of overexpression of rate-limiting genes strategy, multi-copy integration technology effectively bolstered the ability of the recombinant strain to produce chelerythrine, with a yield 224 times higher than that of the first-generation recombinant yeast Z4. Tailoring gene copy numbers to pathway requirements is an efficient strategy for biosynthesis. This approach can also be used to improve the synthesis pathways of other compounds.

Due to the complexity and specificity of its catalytic reactions, optimizing the restriction of P450 enzymes remains a crucial bottleneck for enhancing the efficiency of multi-enzyme artificial pathways [32, 48]. The proper redox partners and adequate attachment space for P450 enzymes significantly enhance their activity [29, 38]. Hence, overexpression of genes encoding redox partners (*PsCPR* and *AtATR1*) and ER expansion factor gene (*INO2*) can effectively increase the chelerythrine titer by 3–8 times (Fig. 3c). In addition, we observed variations in P450 activity sensitivities to different redox partner genes, suggesting that the impacts of these partners on the pathway is distinct. Among them, *AtATR1* showed sensitivity in both rounds, indicating that it is more suitable for enhancing P450s activity during chelerythrine biosynthesis. Enhancing the cofactors supply can significantly improve the catalytic efficiency of P450 enzymes [32]. Overexpression of rate-limiting enzymes in the heme biosynthetic pathway alleviates the stress imposed by P450 enzyme expression and increases the P450 enzyme activity by 2.3-fold [37]. By overexpressing the downstream genes of PPP, combinatorial expression of *XFPK* and *PTA* can enhance the PPP for NADPH regeneration [49]. By optimizing the supply of heme and NADPH, Liu et al. significantly increased the production of bioactive isoflavonoids in an engineered yeast cell factory [31]. Regulating heme biosynthetic pathway and pentose phosphate pathway for increasing heme and NADPH supply drastically enhanced the chelerythrine titer (from 74.74 to 195.03 µg/L) (Figs. 4b and 5b), indicating that improving the cofactor supply is effective for synthesizing target products in engineered yeast cell factories.

This study successfully achieved the first total biosynthesis of chelerythrine in yeast with a significantly higher synthesis efficiency than that of most complex natural plant products. This was achieved through an effective metabolic regulation strategy. To further enhance the synthesis efficiency, we are currently exploring solutions to several bottlenecks, including (1) Inadequate substrate supply. The current substrate being fed requires membrane transport, and an insufficient substrate supply to biosynthetic enzymes might decrease the reaction efficiency. Therefore, it is necessary to introduce the metabolic system of the precursor supply into the engineered yeast to achieve *de novo* synthesis of chelerythrine. (2) The engineering of P450 enzymes is necessary. Despite implementing metabolic engineering strategies such as overexpressing redox partner genes, expanding ER surface area to improve enzyme expression and catalytic sites, increasing the availability of the cofactor heme, and reinforcing reducible NADPH levels, regulating P450s activity is the most significant challenge. A structure-guided mutagenesis library is required to refine the precise structure of the active site, screen for highly active enzyme mutants, and enhance the biosynthesis of natural products. (3) Accumulation of precursors. Although the synthetic flux of chelerythrine was improved using metabolic engineering strategies, the accumulation of precursors compromises the overall yield of chelerythrine. Analyzing the metabolic pathway and identifying more efficient and selective transporters can circumvent the accumulation of precursors and enhance the yield and productivity of chelerythrine. Our research holds great potential to advance this field and create the possibility of commercially producing complex alkaloids via yeast fermentation.

## Conclusions

Using a yeast cell factory, we demonstrated a strategy for the efficient assembly and regulation to the biosynthetic pathway for chelerythrine. Chelerythrine production was significantly enhanced by combining comprehensive strategies, including screening candidate enzymes, integrating multiple-copy rate-limiting genes, optimizing the supply of cofactors, and increasing the transporter that regulate the synthetic efficiency and product transportation. The chelerythrine yield in the best-performing recombinant strain, Z22, reached 12.61 mg/L, representing more than 37,000-fold enhancement compared to the strain Z4 using the simple assembly pathway. This study presents an effective scheme for regulating heterologous pathways in yeast, offering a green alternative that can be readily applied to the efficient synthesis of other valuable compounds in microbial cells.

### Electronic supplementary material

Below is the link to the electronic supplementary material.

Supplementary Material 1

## Data Availability

The datasets used and/or analysed during the current study are available from the corresponding author on reasonable request.

## Abbreviations

Ac-CoA: Acetyl-CoA
Ace: Acetaldehyde
AcP: Acetyl-phosphate
BBE: berberine bridge enzyme
BIAs: benzylisoquinoline alkaloids
CPRs: cytochrome P450 reductases
DBOX: dihydrobenzophenanthridine oxidase
E4P: Erythrose-4-phosphate
ER: endoplasmic reticulum
F6P: Fructose 6-phosphate
G3P: Glyceraldehyde-3-phosphate
G6P: Glucose-6-phosphate
GδL6P: 6-phosphogluconolactone
gRNA: guide RNA
Hac: Acetate
LB: Luria-Bertani
LC-MS: liquid chromatograph-mass spectrometry
LC-MS/MS: liquid chromatography-tandem mass spectrometry
MSH: (S)-cis-N-methylstylopine 14-hydroxylase
OD_600_: optical density at 600 nm
PPP: pentose phosphate pathway
Pyr: Pyruvicacid
P6H: protopine 6-hydroxylase
Ru5p: Ribulose 5-phosphate
R5P: Ribose 5-phosphate
SMT: (S)-scoulerine-9-O-methyltransferase enzyme
S7P: Sedoheptulose 7-phosphate
TDC: (S)-canadine synthase
TNMT: tetrahydroprotoberberine cis-N-methyltransferase
X5P: Xylose 5-phosphate
6PG: 6-phosphogluconate
