# Supplementary material for: Metabolic engineering of Saccharomyces cerevisiae for chelerythrine biosynthesis
Source: Microb Cell Fact. 2024 Jun 21;23:183. doi: 10.1186/s12934-024-02448-4 (PMC11191272; doi:10.1186/s12934-024-02448-4)
Supplement: Supplementary file 1 — Supplementary Material 1 [file 12934_2024_2448_MOESM1_ESM.docx]

**Metabolic engineering of *Saccharomyces cerevisiae* for chelerythrine biosynthesis**

Jiawei Zhu, Kai Zhang, Yuanzhi He, Qi Zhang, Yanpeng Ran, Zaigao Tan,

Li Cui* & Yan Feng*

State Key Laboratory of Microbial Metabolism, School of Life Sciences and Biotechnology, Shanghai Jiao Tong University, Shanghai 200240, China

*Corresponding authors. Address: Shanghai Jiao Tong University, 800 Dongchuan Rd., Shanghai 200240, China. Fax: (86)21-34207189

E-mail address:

[yfeng2009@sjtu.edu.cn](mailto:yfeng2009@sjtu.edu.cn) (Y. Feng);

cuili@sjtu.edu.cn (L. Cui)

**Table S1. Strains and plasmids used in this study.**

| Strain | Genotype | Source |
| --- | --- | --- |
| Z0 | Wild type *S.* *cerevisiae* W303-1A | Our lab |
| *E. coli* Top10 | Wild-type *E. coli* | Our lab |
| Z0′ | ZC-L1::TEF1p-PsCPR-TEF1t | This study |
| Z1 | Z0-L1::TEF1p-tMcoBBE-TEF1t | This study |
| Z2 | Z0-L1::PGK1p-PsSMT-PGK1t+ENO2p-AmTDC-ENO2t+TEF1p-PsCPR-TEF1t | This study |
| Z3 | Z0-L2::PYK2p-EcTNMT-ADH1t+HXT7p-PsMSH-ADH2t+FBA1p-EcP6H-CYC1t+TEF1p-PsCPR-TEF1t+CDC60p-PsDBOX-TDH2t | This study |
| Z3′ | Z0-L2::PYK2p-EcTNMT-ADH1t+HXT7p-PsMSH-ADH2t+FBA1p-EcP6H-CYC1t+TEF1p-PsCPR-TEF1t | This study |
| Z4 | Z3-L1::TEF1p-tMcoBBE-TEF1t+PGK1p-PsSMT-PGK1t+ENO2p-AmTDC-ENO2t | This study |
| Z5 | Z4-L3::PGK1p-PsSMT-PGK1t+ENO2p-AmTDC-ENO2t+PYK2p-EcTNMT-ADH1t+HXT7p-PsMSH-ADH2t+FBA1p-EcP6H-CYC1t+TEF1p-PsCPR-TEF1t+PGK1p-INO2-PGK1t | This study |
| Z6 | Z5-L4::PGK1p-PsSMT-PGK1t+ENO2p-AmTDC-ENO2t+PYK2p-EcTNMT-ADH1t+HXT7p-PsMSH-ADH2t+FBA1p-EcP6H-CYC1t+TEF1p-PsCPR-TEF1t+PGK1p-INO2-PGK1t | This study |
| Z7 | Z6-L5::PGK1p-PsSMT-PGK1t+ENO2p-AmTDC-ENO2t+PYK2p-EcTNMT-ADH1t+HXT7p-PsMSH-ADH2t+FBA1p-EcP6H-CYC1t+TEF1p-PsCPR-TEF1t+PGK1p-INO2-PGK1t | This study |
| Z8 | Z7-L6::PGK1p-PsSMT-PGK1t+ENO2p-AmTDC-ENO2t+PYK2p-EcTNMT-ADH1t+HXT7p-PsMSH-ADH2t+FBA1p-EcP6H-CYC1t+TEF1p-PsCPR-TEF1t+PGK1p-INO2-PGK1t | This study |
| Z9 | Z8-L7::PGK1p-PsSMT-PGK1t+ENO2p-AmTDC-ENO2t+PYK2p-EcTNMT-ADH1t+HXT7p-PsMSH-ADH2t+FBA1p-EcP6H-CYC1t+TEF1p-PsCPR-TEF1t+PGK1p-INO2-PGK1t | This study |
| Z10 | Z9-L8::HXT7p-PsMSH-ADH2t+PGK1p-INO2-PGK1t+TEF1p-AtATR1-TEF1t | This study |
| Z11 | Z10-L9::HXT7p-PsMSH-ADH2t+PGK1p-INO2-PGK1t+TEF1p-AtATR1-TEF1t | This study |
| Z12 | Z11-L10::HXT7p-PsMSH-ADH2t+PGK1p-INO2-PGK1t+TEF1p-AtATR1-TEF1t | This study |
| Z13 | Z12-L11::HXT7p-PsMSH-ADH2t+PGK1p-INO2-PGK1t+TEF1p-AtATR1-TEF1t | This study |
| Z14 | Z13-L12::TEF1p-HEM3-TEF1t+TEF1p-HEM2-CYC1t | This study |
| Z15 | Z14-L13:: THS1p-HEM12-SSD1t | This study |
| Z16 | Z15-*ΔROX1* | This study |
| Z17 | Z16-*ΔHMX1* | This study |
| Z18 | Z17-L14::TEF1p-ZWF1-TEF1t | This study |
| Z19 | Z18-L15::TYS1p-GDN1-TPS1t | This study |
| Z20 | Z19-L16::ALA1p-TKL1-PYC2t+MTS1p-TKL2-APE2t+FRS1p-TAL1-APT15t | This study |
| Z21 | Z20-*ΔGPP1* (L17)::CDC60p-XFPK-ATP15t+  THS1p-PTA-SSD1t | This study |
| Z22 | Z21-L18::TEF1p-MtABCG10-TEF1t | This study |
| Z401 | Z4 harboring plasmid pRS416-tMcoBBE | This study |
| Z402 | Z4 harboring plasmid pRS416-TfSMT | This study |
| Z403 | Z4 harboring plasmid pRS416-AmTDC | This study |
| Z404 | Z4 harboring plasmid pRS416-EcTNMT | This study |
| Z405 | Z4 harboring plasmid pRS416-PsMSH | This study |
| Z406 | Z4 harboring plasmid pRS416-EcP6H | This study |
| Z407 | Z4 harboring plasmid pRS416-INO2 | This study |
| Z408 | Z4 harboring plasmid pRS416-PsCPR | This study |
| Z409 | Z4 harboring plasmid pRS416-AtATR1 | This study |
| Z410 | Z4 harboring plasmid pRS416-AtATR2 | This study |
| Z901 | Z9 harboring plasmid pRS416-tMcoBBE | This study |
| Z902 | Z9 harboring plasmid pRS416-TfSMT | This study |
| Z903 | Z9 harboring plasmid pRS416-AmTDC | This study |
| Z904 | Z9 harboring plasmid pRS416-EcTNMT | This study |
| Z905 | Z9 harboring plasmid pRS416-PsMSH | This study |
| Z906 | Z9 harboring plasmid pRS416-EcP6H | This study |
| Z907 | Z9 harboring plasmid pRS416-INO2 | This study |
| Z908 | Z9 harboring plasmid pRS416-PsCPR | This study |
| Z909 | Z9 harboring plasmid pRS416-AtATR1 | This study |
| Z171 | Z17 harboring plasmid pRS416-ALD6 | This study |
| Z211 | Z21-L18::TEF1p-McoABC-TEF1t | This study |
| Z212 | Z21-L18::TEF1p-CrTPT-TEF1t | This study |
| Z213 | Z21-L18::TEF1p-CjABCB2-TEF1t | This study |
| Z0(AmBBE) | Z0 harboring plasmid pRS416-AmBBE | This study |
| Z0(EcBBE) | Z0 harboring plasmid pRS416-EcBBE | This study |
| Z0(McoBBE) | Z0 harboring plasmid pRS416-McoBBE | This study |
| Z0(tMcoBBE) | Z0 harboring plasmid pRS416-tMcoBBE | This study |
| Z0(PsBBE) | Z0 harboring plasmid pRS416-PsBBE | This study |
| Z0(AmSMT) | Z0 harboring plasmid pRS416-AmSMT | This study |
| Z0(CcSMT) | Z0 harboring plasmid pRS416-CcSMT | This study |
| Z0(PsSMT) | Z0 harboring plasmid pRS416-PsSMT | This study |
| Z0(TfSMT) | Z0 harboring plasmid pRS416-TfSMT | This study |
| Z0(CmTNMT) | Z0 harboring plasmid pRS416-CmTNMT | This study |
| Z0(EcTNMT) | Z0 harboring plasmid pRS416-EcTNMT | This study |
| Z0(GfTNMT) | Z0 harboring plasmid pRS416-GfTNMT | This study |
| Z0(PsTNMT) | Z0 harboring plasmid pRS416-PsTNMT | This study |
| Z0′(EcP6H) | Z0′ harboring plasmid pRS416-EcP6H | This study |
| Z0′(t4EcP6H) | Z0′ harboring plasmid pRS416-t4EcP6H | This study |
| Z0′(t27EcP6H) | Z0′ harboring plasmid pRS416-t27EcP6H | This study |
| Z0′(McoP6H) | Z0′ harboring plasmid pRS416-McoP6H | This study |
| Z0′(PsP6H) | Z0′ harboring plasmid pRS416-PsP6H | This study |
| Z0(GFP-McoBBE) | Z0 harboring plasmid pRS416-GFP-McoBBE | This study |
| Z0(GFP-tMcoBBE) | Z0 harboring plasmid pRS416-GFP-tMcoBBE | This study |
| Z0(GFP-AmSMT) | Z0 harboring plasmid pRS416-GFP-AmSMT | This study |
| Z0(GFP-AmTDC) | Z0 harboring plasmid pRS416-GFP-AmTDC | This study |
| Z0(GFP-PsTNMT) | Z0 harboring plasmid pRS416-GFP-PsTNMT | This study |
| Z0(GFP-PsMSH) | Z0 harboring plasmid pRS416-GFP-PsMSH | This study |
| Z0(GFP-PsP6H) | Z0 harboring plasmid pRS416-GFP-PsP6H | This study |
| Z0(GFP-McoDBOX) | Z0 harboring plasmid pRS416-GFP-McoDBOX | This study |
| Z0(GFP-PsCPR) | Z0 harboring plasmid pRS416-GFP-PsCPR | This study |
| pRS416 | Expression vector, Ap^R^ | Our lab |
| pRS416-AmBBE | pRS416 containing P_TEF1_-AmBBE-T_TEF1_ cassette | This study |
| pRS416-EcBBE | pRS416 containing P_TEF1_-EcBBE-T_TEF1_ cassette | This study |
| pRS416-McoBBE | pRS416 containing P_TEF1_-McoBBE-T_TEF1_ cassette | This study |
| pRS416-tMcoBBE | pRS416 containing P_TEF1_-tMcoBBE-T_TEF1_ cassette | This study |
| pRS416-PsBBE | pRS416 containing P_TEF1_-PsBBE-T_TEF1_ cassette | This study |
| pRS416-AmSMT | pRS416 containing P_PGK1_-AmSMT-T_PGK1_ cassette | This study |
| pRS416-CcSMT | pRS416 containing P_PGK1_-CcSMT-T_PGK1_ cassette | This study |
| pRS416-PsSMT | pRS416 containing P_PGK1_-PsSMT-T_PGK1_ cassette | This study |
| pRS416-TfSMT | pRS416 containing P_PGK1_-TfSMT-T_PGK1_ cassette | This study |
| pRS416-CmTNMT | pRS416 containing P_PYK2_-CmTNMT-T_ADH1_ cassette | This study |
| pRS416-EcTNMT | pRS416 containing P_PYK2_-EcTNMT-T_ADH1_ cassette | This study |
| pRS416-GfTNMT | pRS416 containing P_PYK2_-GfTNMT-T_ADH1_ cassette | This study |
| pRS416-PsTNMT | pRS416 containing P_PYK2_-PsTNMT-T_ADH1_ cassette | This study |
| pRS416-EcP6H | pRS416 containing P_FBA1_-EcP6H-T_CYC1_ cassette | This study |
| pRS416-t4EcP6H | pRS416 containing P_FBA1_-t4P6H-T_CYC1_ cassette | This study |
| pRS416-t27EcP6H | pRS416 containing P_FBA1_-t27P6H-T_CYC1_ cassette | This study |
| pRS416-McoP6H | pRS416 containing P_FBA1_-McoP6H-T_CYC1_ cassette | This study |
| pRS416-PsP6H | pRS416 containing P_FBA1_-PsP6H-T_CYC1_ cassette | This study |
| pRS416-AmTDC | pRS416 containing P_ENO2_-AmTDC-T_ENO2_ cassette | This study |
| pRS416-PsMSH | pRS416 containing P_HXT7_-PsMSH-T_ADH2_ cassette | This study |
| pRS416-PsCPR | pRS416 containing P_TEF1_-PsCPR-T_TEF1_ cassette | This study |
| pRS416-AtATR1 | pRS416 containing P_TEF1_-AtATR1-T_TEF1_ cassette | This study |
| pRS416-AtATR2 | pRS416 containing P_TEF1_-AtATR2-T_TEF1_ cassette | This study |
| pRS416-INO2 | pRS416 containing P_PGK1_-INO2-T_PGK1_ cassette | This study |
| pRS416-ALD6 | pRS416 containing P_TEF1_-ALD6-T_TEF1_ cassette | This study |
| pRS416-GFP-McoBBE | pRS416 containing P_TEF1_-GPF-McoBBE-T_TEF1_ cassette | This study |
| pRS416-GFP-tMcoBBE | pRS416 containing P_TEF1_-GFP-tMcoBBE-T_TEF1_ cassette | This study |
| pRS416-GFP-AmSMT | pRS416 containing P_PGK1_-GFP-AmSMT-T_PGK1_ cassette | This study |
| pRS416-GFP-AmTDC | pRS416 containing P_ENO2_-GFP-AmTDC-T_ENO2_ cassette | This study |
| pRS416-GFP-PsTNMT | pRS416 containing P_PYK2_-GFP-PsTNMT-T_ADH1_ cassette | This study |
| pRS416-GFP-PsMSH | pRS416 containing P_HXT7_-GFP-PsMSH-T_ADH2_ cassette | This study |
| pRS416-GFP-PsP6H | pRS416 containing P_FBA1_-GFP-PsP6H-T_CYC1_ cassette | This study |
| pRS416-GFP-McoDBOX | pRS416 containing P_CDC60_-GFP-McoDBOX-T_TDH1_ cassette | This study |
| pRS416-GFP-PsCPR | pRS416 containing P_TEF1_-GFP-PsCPR-T_TEF1_ cassette | This study |
| p414-TEF1p-Cas9-CYC1t | Cas9 expression plasmid | Our lab |
| p426-SNR52p-gRNA.CAN1.Y-SUP4t | Guide RNA expression plasmid targeting to insert modules A or/and B | Our lab |
| gRNA-Z3 | gRNA plasmid targeting to insert module C | This study |
| gRNA-Z5 | gRNA plasmid targeting to insert cassette A | This study |
| gRNA-Z6 | gRNA plasmid targeting to insert cassette A | This study |
| gRNA-Z7 | gRNA plasmid targeting to insert cassette A | This study |
| gRNA-Z8 | gRNA plasmid targeting to insert cassette A | This study |
| gRNA-Z9 | gRNA plasmid targeting to insert cassette A | This study |
| gRNA-Z10 | gRNA plasmid targeting to insert cassette B | This study |
| gRNA-Z11 | gRNA plasmid targeting to insert cassette B | This study |
| gRNA-Z12 | gRNA plasmid targeting to insert cassette B | This study |
| gRNA-Z13 | gRNA plasmid targeting to insert cassette B | This study |
| gRNA-Z14 | gRNA plasmid targeting to insert *HEM2*+*HEM3* cassette | This study |
| gRNA-Z15 | gRNA plasmid targeting to insert *HEM12* cassette | This study |
| gRNA-Z16 | gRNA plasmid targeting to knock out the *ROX1* gene and insert *ZWF1* cassette | This study |
| gRNA-Z17 | gRNA plasmid targeting to knock out the *HMX1* gene and insert *GND1* cassette | This study |
| gRNA-Z19 | gRNA plasmid targeting to insert *TAL1*+*TKL1*+*TKL2* cassette | This study |
| gRNA-Z20 | gRNA plasmid targeting to knock out the *GPP1* gene and insert *LmXFPK*+*CkPTA* cassette | This study |
| gRNA-Z22 | gRNA plasmid targeting to insert *MtABCG10* or other transporters cassette | This study |

**Table S2. Integration sites utilized in this study.**

| *S. cerevisiae* locus | Corresponding gRNA | Cas9 target site* |
| --- | --- | --- |
| L1 | p426-SNR52p-gRNA.CAN1.Y-SUP4t | GATACGTTCTCTATGGAGGATGG |
| L2 | gRNA-Z3 | CCAGAGTGTATAAAATGTGGCGG |
| L3 | gRNA-Z5 | TGTACTTATTAAAGTAGAAGTGG |
| L4 | gRNA-Z6 | TGTACCAAAAGTTATCCTGTAGG |
| L5 | gRNA-Z7 | ATAGAATTACTATTGAAGAGTGG |
| L6 | gRNA-Z8 | ATAATTAATGTTGAACCAATCGG |
| L7 | gRNA-Z9 | GACAGCATATTAAACAGAAGAGG |
| L8 | gRNA-Z10 | AAATCCTATACGATGAAGTATGG |
| L9 | gRNA-Z11 | TTAAGGACAGAATATTAAACAGG |
| L10 | gRNA-Z12 | CCTATTGGACAAGATTTACGAGG |
| L11 | gRNA-Z13 | GTTAGAGCTGTTACAAGTTACGG |
| L12 | gRNA-Z14 | TATCCTGAATGTTCTCTCCCAGG |
| L13 | gRNA-Z15 | TGAGAAACGGCTATCGGATGTGG |
| L14 | gRNA-Z16 | GGTGTGGGTTTAGATGACAAGGG |
| L15 | gRNA-Z17 | ACCGGAAGATAAGGCACACTGGG |
| L16 | gRNA-Z19 | CATCCACGAAAACATACACAAGG |
| L17 | gRNA-Z20 | CTTAAAGGGTAGAAACGGTTTGG |
| L18 | gRNA-Z22 | TTGTATAATATTGAAAATAAAGG |

***** PAMs are underlined.

**Table S3.** **Heterologous genes used in this study**.

| Gene/Enzyme | Source | NCBI Accession No.  (Amino acid sequence) |
| --- | --- | --- |
| *AmBBE*/AmBBE | *Argemone mexicana* | ACJ76783.1 |
| *EcBBE*/EcBBE | *Eschscholzia californica* | P30986.1 |
| *McoBBE*/McoBBE | *M. cordata* | MK376320.1 |
| *PsBBE*/PsBBE | *Papaver somniferum* | XM_026566048.1 |
| *AmSMT*/AmSMT | *A. mexicana* | KT984756.1 |
| *CcSMT*/CcSMT | *Copti. chinensis* | EU980450.1 |
| *PsSMT*/PsSMT | *P. somniferum* | JN185323.1 |
| *TfSMT*/TfSMT | *Thalictrum flavum* subsp. glaucum | AY610512.1 |
| *CmTNMT*/CmTNMT | *Chelidonium majus* | MK424053.1 |
| *EcTNMT*/EcTNMT | *E. californica* | EU882977.1 |
| *GfTNMT*/GfTNMT | *Glaucium flavum* | 6P3M_A |
| *PsTNMT*/PsTNMT | *P. somniferum* | DQ028579.1 |
| *EcP6H*/EcP6H | *E. californica* | AB598834.1 |
| *McoP6H*/McoP6H | *M. cordata* | OVA18597.1 |
| *PsP6H*/PsP6H | *P. somniferum* | KC154002.1 |
| *AmTDC*/AmTDC | *A. mexicana* | B1NF19.1 |
| *PsMSH*/PsMSH | *P. somniferum* | KC154003.1 |
| *McoDBOX*/McoDBOX | *M. cordata* | OVA00267.1 |
| *PsCPR*/PsCPR | *P. somniferum* | AAC05021.1 |
| *AtATR1*/AtATR1 | *Arabidopsis thaliana* | NP_194183.1 |
| *AtATR2*/AtATR2 | *A. thaliana* | NP_194750.1 |
| *MtABCG10*/MtABCG10 | *Medicago truncatula* | AES68070.1 |
| *McoABC*/McoABC | *M. cordata* | OUZ99660.1 |
| *CjABCB2*/CjABCB2 | *Coptis japonica* | AB674325.1 |
| *CrTPT2*/CrTPT2 | *Catharanthus roseus* | KAI5669436.1 |
| *LmXFPK*/LmXFPK | *Leuconostoc mesenteroides* | CP065995.1 |
| *CkPTA*/CkPTA | *Clostridium kluyveri* | AP009049.1 |

**Table S4. List of oligonucleotides used in this study.**

| Primer | Sequence (5’-3’) |
| --- | --- |
| Construction of pRS416-AmBBE plasmid | |
| TEF1p-AmBBE-F | AGCAATCTAATCTAAGTTTTAATTACAAAATGTTGACTACTGTTACTATGG |
| TEF1t-AmBBE-R | ACTAGAAAAGTCTTATCAATCTCCTTAGAAGATAACGTTACCTTGGTC |
| AmBBE-TEF1p-R | AGTTTCCATAGTAACAGTAGTCAACATTTTGTAATTAAAACTTAGATTAGATTG |
| AmBBE-TEF1t-F | ACCAAGGTAACGTTATCTTCTAAGGAGATTGATAAGACTTTTCTAG |
| XhoI-TEF1p-F | CCGCTCGAGCGGATAGCTTCAAAATGTTTCTACTC |
| BamHI-TEF1t-R | CGCGGATCCGCGATGGCTTCCCCGCTATACTTAAG |
| Construction of pRS416-EcBBE plasmid | |
| TEF1p-EcBBE-F | AGCAATCTAATCTAAGTTTTAATTACAAAATGGAAAACAAGACTCCAATCTTCTTC |
| TEF1t-EcBBE-R | ACTAGAAAAGTCTTATCAATCTCCTTAGATAACAACTTCACCACCGTCAG |
| EcBBE-TEF1p-R | AAGAAGATTGGAGTCTTGTTTTCCATTTTGTAATTAAAACTTAGATTAGATTG |
| EcBBE-TEF1t-F | ACGGTGGTGAAGTTGTTATCTAAGGAGATTGATAAGACTTTTCTAG |
| Construction of pRS416-McoBBE plasmid | |
| TEF1p-McoBBE-F | AGCAATCTAATCTAAGTTTTAATTACAAAATGGAAAACAAGACTCCAATCTTC |
| TEF1t-McoBBE-R | ACTAGAAAAGTCTTATCAATCTCCTTAGATAACAACTTCACCACCGTCAG |
| McoBBE-TEF1p-R | AGAAGATTGGAGTCTTGTTTTCCATTTTGTAATTAAAACTTAGATTAGATTG |
| McoBBE-TEF1t-F | ACGGTGGTGAAGTTGTTATCTAAGGAGATTGATAAGACTTTTCTAG |
| Construction of pRS416-tMcoBBE plasmid | |
| TEF1p-tMcoBBE-F | AGCAATCTAATCTAAGTTTTAATTACAAAATGGACGATTTGTTGTCTTGTTTGACTTC |
| tMcoBBE-TEF1p-R | AGAAGTCAAACAAGACAACAAATCGTCCATTTTGTAATTAAAACTTAGATTAGATTG |
| Construction of pRS416-PsBBE plasmid | |
| TEF1p-PsBBE-F | AGCAATCTAATCTAAGTTTTAATTACAAAATGATGTGTAGATCTTTGACTTTGAG |
| TEF1t-PsBBE-R | ACTAGAAAAGTCTTATCAATCTCCTTACAATTCCTTCAACATGTAGATTTC |
| PsBBE-TEF1p-R | ATCTCAAAGTCAAAGATCTACACATCATTTTGTAATTAAAACTTAGATTAGATTG |
| PsBBE-TEF1t-F | AATCTACATGTTGAAGGAATTGTAAGGAGATTGATAAGACTTTTCTAG |
| Construction of pRS416-GFP-McoBBE plasmid | |
| GFP-TEF1p-R | TCTCCTCGCCCTTGGACACCATTTTGTAATTAAAACTTAGATTAG |
| TEF1p-GFP-F | TAATCTAAGTTTTAATTACAAAATGGTGTCCAAGGGCGAGGAGAC |
| McoBBE-GFP-R | AGTTTCTAATCTTAGTATCCATCTTATACAGCTCGTCCATG |
| GFP-McoBBE-F | ACGGCATGGACGAGCTGTATAAGATGGATACTAAGATTAGAAAC |
| Construction of pRS416-GFP-tMcoBBE plasmid | |
| GFP-tMcoBBE-F | ACGGCATGGACGAGCTGTATAAGGACGATTTGTTGTCTTGTTTGACTTCTCAC |
| tMcoBBE-GFP-R | AGAAGTCAAACAAGACAACAAATCGTCCACATCTTATACAGCTCGTCCATG |
| Construction of pRS416-GFP-AmSMT plasmid | |
| GFP-AmSMT-F | ACGGCATGGACGAGCTGTATAAGATGGATGTTAACAACGGTATTGTTG |
| AmSMT-GFP-R | AACAATACCGTTGTTAACATCTTATACAGCTCGTCCATG |
| PGK1p-GFP-F | ACTTTTTACAACAAATATAAAAACAATGGTGTCCAAGGGCGAGGAGAC |
| GFP-PGK1p-R | TCTCCTCGCCCTTGGACACCATCCATTGTTTTTATATTTGTTGTAAAAAGTAG |
| Construction of pRS416-GFP-AmTDC plasmid | |
| GFP-AmTDC-F | ACGGCATGGACGAGCTGTATAAGATGGAAGAAAAAATCATGAC |
| AmTDC-GFP-R | GTCATGATTTTTTCTTCCATCTTATACAGCTCGTCCATG |
| ENO2p-GFP-F | CTAATACTATAACATACAATAATAATGGTGTCCAAGGGCGAGGAGAC |
| GFP-ENO2p-R | TCTCCTCGCCCTTGGACACCATTATTATTGTATGTTATAGTAT |
| Construction of pRS416-GFP-PsTNMT plasmid | |
| GFP-PsTNMT-F | ACGGCATGGACGAGCTGTATAAGATGGGTTCTATTGATGAAGTTAAG |
| PsTNMT-GFP-R | TCTTAACTTCATCAATAGAACCCATCTTATACAGCTCGTCCATG |
| PYK2p-GFP-F | AAGATTACAACAAAAGCACTATCGATGGTGTCCAAGGGCGAGGAGAC |
| GFP-PYK2p-R | TCTCCTCGCCCTTGGACACCATCGATAGTGCTTTTGTTGTAATCTTAC |
| Construction of pRS416-GFP-PsMSH plasmid | |
| GFP-PsMSH-F | ACGGCATGGACGAGCTGTATAAGATGAGAACTGAATCTATAAAAAC |
| PsMSH-GFP-R | TTTATAGATTCAGTTCTCATCTTATACAGCTCGTCCATG |
| HXT7p-GFP-F | CATACCCCTCATTTCCACGGGATGGTGTCCAAGGGCGAGGAGAC |
| GFP-HXT7p-R | TCTCCTCGCCCTTGGACACCATCCCGTGGAAATGAGGGGTATG |
| Construction of pRS416-GFP-PsP6H plasmid | |
| GFP-PsP6H-F | ACGGCATGGACGAGCTGTATAAGATGGATTTCTCTTCTTTGTTGTTGTTG |
| PsP6H-GFP-R | ACAACAACAAAGAAGAGAAATCCATCTTATACAGCTCGTCCATG |
| FBA1p-GFP-F | ACCATAACCAAGTAATACATATTCAAAATGGTGTCCAAGGGCGAGGAGAC |
| GFP-FBA1p-R | TCTCCTCGCCCTTGGACACCATTTTGAATATGTATTACTTGGTTATG |
| Construction of pRS416-GFP-McoDBOX plasmid | |
| GFP-McoDBOX-F | ACGGCATGGACGAGCTGTATAAGATGGCAGATTCATCTAAAAAGCT |
| McoDBOX-GFP-R | AGCTTTTTAGATGAATCTGCCATCTTATACAGCTCGTCCATG |
| CDC60p-GFP-F | ATATCTTTAACCAGTCTATAATGGTGTCCAAGGGCGAGGAGAC |
| GFP-CDC60p-R | TCTCCTCGCCCTTGGACACCATTATAGACTGGTTAAAGATAT |
| Construction of pRS416-GFP-PsCPR plasmid | |
| GFP-PsCPR-F | ACGGCATGGACGAGCTGTATAAGATGGGTTCTAACAACTTGGC |
| PsCPR-GFP-R | GCCAAGTTGTTAGAACCCATCTTATACAGCTCGTCCATG |
| Construction of pRS416-AmSMT plasmid | |
| XhoI-PGK1p-F | CCGCTCGAGAAAGATGCCGATTTGGGCGCGAATC |
| AmSMT-PGK1p-R | AACAATACCGTTGTTAACATCCATTGTTTTTATATTTGTTGTAAAAAGTAG |
| PGK1p-AmSMT-F | ACTTTTTACAACAAATATAAAAACAATGGATGTTAACAACGGTATTGTTG |
| PGK1t-AmSMT-R | ATCTATCGATTTCAATTCAATTCTTAATCATGCAAAACCCAGTTCAAC |
| AmSMT-PGK1t-F | TGTTGAACTGGGTTTTGCATGATTAAGAATTGAATTGAAATCGATAGATC |
| BamHI-PGK1t-R | GCGGGATCCTTTTGTTGCAAGTGGGATGAGCTTG |
| Construction of pRS416-CcSMT plasmid | |
| CcSMT-PGK1p-R | AGTTAACACCTTCTTGAGCTGCCATTGTTTTTATATTTGTTGTAAAAAGTAG |
| PGK1p-CcSMT-F | ACTTTTTACAACAAATATAAAAACAATGGCAGCTCAAGAAGGTGTTAAC |
| PGK1t-CcSMT-R | ATCTATCGATTTCAATTCAATTCTTAACAGTTAATTTTATGAAATTCC |
| CcSMT-PGK1t-F | ATGGAATTTCATAAAATTAACTGTTAAGAATTGAATTGAAATCGATAGATC |
| Construction of pRS416-PsSMT plasmid | |
| PsSMT- PGK1p-R | TGTTAAAAATTTCACCGTTAGTAGCCATTGTTTTTATATTTGTTGTAAAAAGTAG |
| PGK1p- PsSMT-F | ACTTTTTACAACAAATATAAAAACAATGGCTACTAACGGTGAAATTTTTAAC |
| PGK1t-PsSMT-R | ATCTATCGATTTCAATTCAATTCTTACTTATGGAATTCAATAACGTG |
| PsSMT-PGK1t-F | ACGTTATTGAATTCCATAAGTAAGAATTGAATTGAAATCGATAGATC |
| Construction of pRS416-TfSMT plasmid | |
| TfSMT-PGK1p-R | AGTTAACACCTTCTTGCAATGCCATTGTTTTTATATTTGTTGTAAAAAGTAG |
| PGK1p-SMT-F | ACTTTTTACAACAAATATAAAAACAATGGCATTGCAAGAAGGTGTTAAC |
| PGK1t-TfSMT-R | ATCTATCGATTTCAATTCAATTCTTAAGATGCAACACCAGCAGTAG |
| TfSMT-PGK1t-F | AGCTACTGCTGGTGTTGCATCTTAAGAATTGAATTGAAATCGATAGATC |
| Construction of pRS416-CmTNMT plasmid | |
| XhoI-PYK2p-F | CCGCTCGAGCGCTTTTATGAACATATTCCGATATC |
| CmTNMT-PYK2p-R | ATTGATGTCTAACTAATTTAGACATCGATAGTGCTTTTGTTGTAATCTTAC |
| PYK2p-CmTNMT-F | AAGATTACAACAAAAGCACTATCGATGTCTAAATTAGTTAGACATCAATGG |
| ADH1t-CmTNMT-R | TAAAAATCATAAATCATAAGAAATTCGCTTACTTATCTAACTTTTTTCTCCATTC |
| CmTNMT-ADH1t-F | AATGGAGAAAAAAGTTAGATAAGTAAGCGAATTTCTTATGATTTATG |
| HindIII-ADH1t-R | GGGAAGCTTCTTGAAGCTCAGGTAAGGGG |
| Construction of pRS416-EcTNMT plasmid | |
| BamHI-ADH1t-F | GCGGGATCCCTTGAAGCTCAGGTAAGGGG |
| EcTNMT-PYK2p-R | ATAATTTCACCAGCAGAAGAACCCATCGATAGTGCTTTTGTTGTAATCTTAC |
| PYK2p-EcTNMT-F | AAGATTACAACAAAAGCACTATCGATGGGTTCTTCTGCTGGTGAAATTATG |
| ADH1t-EcTNMT-R | TAAAAATCATAAATCATAAGAAATTCGCTTATTTTTTCTTAAACAAAATTTGAG |
| EcTNMT-ADH1t-F | ACTCAAATTTTGTTTAAGAAAAAATAAGCGAATTTCTTATGATTTATG |
| Construction of pRS416-GfTNMT | |
| GfTNMT-PYK2p-R | ATGATGATGGTGAGAAGAACCCATCGATAGTGCTTTTGTTGTAATCTTAC |
| PYK2p-GfTNMT-F | AAGATTACAACAAAAGCACTATCGATGGGTTCTTCTCACCATCATCATC |
| ADH1t-GfTNMT-R | TAAAAATCATAAATCATAAGAAATTCGCTTATTTCTTTTTAAACAACATTTG |
| GfTNMT-ADH1t-F | TGCTCAAATGTTGTTTAAAAAGAAATAAGCGAATTTCTTATGATTTATG |
| Construction of pRS416-PsTNMT plasmid | |
| PsTNMT-PYK2p-R | TCTTAACTTCATCAATAGAACCCATCGATAGTGCTTTTGTTGTAATCTTAC |
| PYK2p-PsTNMT-F | AAGATTACAACAAAAGCACTATCGATGGGTTCTATTGATGAAGTTAAG |
| ADH1t-PsTNMT-R | TAAAAATCATAAATCATAAGAAATTCGCTTACTTCTTCTTGAACAACAATTG |
| PsTNMT-ADH1t-F | AATTGTTGTTCAAGAAGAAGTAAGCGAATTTCTTATGATTTATG |
| Construction of pRS416-EcP6H plasmid | |
| XhoI-FBA1p-F | CCGCTCGAGATAACAATACTGACAGTACTAAATAATTG |
| FBA1-EcP6H-F | ACCATAACCAAGTAATACATATTCAAAATGGATTCTTTGATGTTGGCTTACTTG |
| EcP6H-FBA1-R | AAGTAAGCCAACATCAAAGAATCCATTTTGAATATGTATTACTTGGTTATG |
| EcP6H-CYC1t-F | ATTACATTATAAGTTGTATGAATAAACAGGCCCCTTTTCCTTTGTCG |
| CYC1t-EcP6H-R | ATGATATCGACAAAGGAAAAGGGGCCTGTTTATTCATACAACTTATAATGTAATCTAG |
| BamHI-CYC1t-F | GCGGGATCCTGGAATGTGAGGGAACAACAAAAG |
| Construction of pRS416-t4EcP6H plasmid | |
| FBA1-t4EcP6H-F | ACCATAACCAAGTAATACATATTCAAAATGATGTTGGCTTACTTGTTCCCAATTTCTG |
| t4EcP6H-FBA1-R | AGAAATTGGGAACAAGTAAGCCAACATCATTTTGAATATGTATTACTTGGTTATG |
| Construction of pRS416-t27EcP6H plasmid | |
| FBA1-t27EcP6H-F | ACCATAACCAAGTAATACATATTCAAAATGTCTTCTAGAACTTTGAAGAACAAGAAG |
| t27EcP6H-FBA1-R | ATCTTCTTGTTCTTCAAAGTTCTAGAAGACATTTTGAATATGTATTACTTGGTTATG |
| Construction of pRS416-PsP6H plasmid | |
| FBA1-PsP6H-F | ACCATAACCAAGTAATACATATTCAAAATGGATTTCTCTTCTTTGTTGTTGTTG |
| PsP6H-FBA1-R | ACAACAACAAAGAAGAGAAATCCATTTTGAATATGTATTACTTGGTTATG |
| PsP6H-CYC1t-F | ATCCTGAATTGTATGATTGTGAAACTTAAACAGGCCCCTTTTCCTTTGTCG |
| CYC1t-PsP6H-R | ATGATATCGACAAAGGAAAAGGGGCCTGTTTAAGTTTCACAATCATACAATTCAG |
| Construction of pRS416-AmTDC plasmid | |
| TDC-ENO2p-R | GTCATGATTTTTTCTTCCATTATTATTGTATGTTATAGTAT |
| ENO2t-TDC-R | TTAGTTAAAAGCACTTTACAATTACATTCTTGGAACAATACCG |
| TDC-ENO2t-F | GGTATTGTTCCAAGAATGTAATTGTAAAGTGCTTTTAACTAAG |
| ENO2p-TDC-F | CTAATACTATAACATACAATAATAATGGAAGAAAAAATCATGAC |
| XhoI-TDC-F | CCGCTCGAGTGCTTATTTTTTCATCATAGTTTAG |
| BamHI-TDC-R | GCGGGATCCTAAAATCAGTCCAGGCAGGGTC |
| Construction of pRS416-PsMSH plasmid | |
| XhoI-MSH-F | CCGCTCGAGAATTTCTAATCTAGGCTTAATC |
| BamHI-MSH-R | CGGGATCCCGGTAGAAGAAATCCAACAAAG |
| MSH-HXT7p-R | TTTATAGATTCAGTTCTCATCCCGTGGAAATGAGGGGTATG |
| HXT7p-MSH-F | CATACCCCTCATTTCCACGGGATGAGAACTGAATCTATAAAAAC |
| ADH2t-MSH-R | TAAAGACATAAGAGATCCGCTTAAATTTCTAATCTAGGCTTAATC |
| MSH-ADH2t-F | AGCCTAGATTAGAAATTTAAGCGGATCTCTTATGTCTTTAC |
| Construction of pRS416-PsCPR plasmid | |
| CPR-TEF1p-R | GCCAAGTTGTTAGAACCCATTTTGTAATTAAAACTTAGATTAG |
| TEF1p-CPR-F | TAATCTAAGTTTTAATTACAAAATGGGTTCTAACAACTTGGC |
| TEF1t-CPR-R | TAGAAAAGTCTTATCAATCTCCTTACCAAACATCTCTCAAGTATC |
| CPR-TEF1t-F | GATACTTGAGAGATGTTTGGTAAGGAGATTGATAAGACTTTTCTAG |
| Construction of pRS416-ALD6 plasmid | |
| TEF1t-ALD6-R | AGAAAAGTCTTATCAATCTCCTTACAACTTAATTCTGACAGCT |
| ALD6-TEF1p-R | TCAAAGTGTAGCTTAGTCATTTTGTAATTAAAACTTAGATTAG |
| TEF1p-ALD6-F | ATCTAAGTTTTAATTACAAAATGACTAAGCTACACTTTGACAC |
| ALD6-TEF1t-F | AGCTGTCAGAATTAAGTTGTAAGGAGATTGATAAGACTTTTC |
| Construction of pRS416-INO2 plasmid | |
| BamHI-PGK1t-R | CGGGGATCCTTTTGTTGCAAGTGGGATGAG |
| PGK1p-INO2-F | TTACAACAAATATAAAACAATGCAACAAGCAACTGGGAAC |
| PGK1t-INO2-R | TATCGATTTCAATTCAATTCTCAGGAATCATCCAGTATGTG |
| INO2-PGK1P-R | GTTCCCAGTTGCTTGTTGCATTGTTTTATATTTGTTGTAAAAAG |
| INO2-PGK1t-F | ACATACTGGATGATTCCTGAGAATTGAATTGAAATCGATAG |
| XhoI-PGK1p-F | CCGCTCGAGACTGTAATTGCTTTTAGTTGTG |
| Construction of pRS416-AtATR1 plasmid | |
| AtATR1-TEF1p-R | AAGCGTACAAAGCGGAGGTCATTTTGTAATTAAAACTTAGATTAG |
| TEF1p-AtATR1-F | TAATCTAAGTTTTAATTACAAAATGACCTCCGCTTTGTACGCTTC |
| AtATR1-TEF1t-F | ATACTTGAGAGATGTTTGGTAAGGAGATTGATAAGACTTTTCTAG |
| TEF1t-AtATR1-R | ACTAGAAAAGTCTTATCAATCTCCTTACCAAACATCTCTCAAGTATC |
| Construction of pRS416-AtATR2 plasmid | |
| AtATR2-TEF1p-R | CACGTTTTGAATTCCCAGAACCGGATCTCATTTTGTAATTAAAACTTAGATTAG |
| TEF1p-AtATR2-F | TCTAATCTAAGTTTTAATTACAAAATGAGATCCGGTTCTGGGAATTCAAAACGTGTC |
| AtATR2-TEF1t-F | AGATATCTTAGAGATGTATGGTAAGGAGATTGATAAGACTTTTCTAG |
| TEF1t-AtATR2-R | ACTAGAAAAGTCTTATCAATCTCCTTACCATACATCTCTAAGATATC |
| Construction of gene expression cassettes of Module A | |
| TYB-Z1-R | AGCTTCACAAACACACCACAGACGTGGGTCAATACCATTGAAAGATGAGAAAAGTAAAGAATTGTATCCATTGCGCTCTTTCCCGACGAGAGTAAATGGCGAGGCGATGGCTTCCCCGCTATACTTAAG |
| TYB-Z1-F | ACCTGTACCAATAGTACCACCAAGGGCAATCATACCAATATGTCTTTGCTTAAGCTCTCTCTTCACTTCAGCGTTCTGTACTTCTCCTTCATCTTCATCACCTATGCCACGGATAGCTTCAAAATGTTTCTACTC |
| Construction of gene expression cassettes of Module B | |
| TYB-PGK1p-F | ATAAATAAATATGATATAAGAGCGCCCACTGGGCCGGCGTTGGTCAGAGGTGTGGATAAACCAATGAAAAGACCTGTACCAATAGTACCACCAAGGGCAATCATACCAATATGTCTTTGCACTGTAATTGCTTTTAGTTGTGTATTTTAGATTCCTGACTTCAACTCAAGACG |
| PGK1t-ENO2p-R | GGATTCCACTTTCCGCAAGTTGGTGCACGTCGTTAGTGACATAACGCCGCGTTTTTGTTGCAAGTGGGATGAGCTTGGAGCAGGAAGAATACACTATACTGG |
| PGK1t-ENO2p-F | ATCCAGTATAGTGTATTCTTCCTGCTCCAAGCTCATCCCACTTGCAACAAAAACGCGGCGTTATGTCACTAACGACGTGCACCAACTTGCGGAAAGTGGAATC |
| TEF1p-ENO2t-R | TAATCTAAGTTTTAATTACAAAATGGGTTCTAACAACTTGGC |
| ENO2t-TEF1p-F | GTGTGTCGTCTTTGAATGGTGGCATATCAAGACCCTGCCTGGACTGATTTTAATAGCTTCAAAATGTTTCTACTCCTTTTTTACTCTTCCAGATTTTCTCGGAC |
| Construction of gene expression cassettes of Module A+B | |
| TEF1t-PGK1p-F | ATCTCTCTTTTACGTAAAGCCTGCGAGATCCTCTTAAGTATAGCGGGGAAGCCATTATTTTAGATTCCTGACTTCAACTCAAGACGCACAGATATTATAACATCTG |
| PGK1p-TEF1t-R | AGATGTTATAATATCTGTGCGTCTTGAGTTGAAGTCAGGAATCTAAAATAATGGCTTCCCCGCTATACTTAAGAGGATCTCGCAGGCTTTACGTAAAAG |
| Construction of gene expression cassettes of Module C | |
| PYK2p-F | ACATACAACTTTTTAAACTAATATACACATTTTAGCAGATGCGCGCACCTGCGTTGTTACCACAACTCTTATGAGGCCCGCGGACAGCATCAAACTGTAAGATTCCGCGCTTTTATGAACATATTCCGATATC |
| HXT7p-ADH1t-R | CAACTTGTCTTTTCTAAGAACAAAGAATAAACACAAAAACAAAAAGTTTTTTTAATTTTAATCAAAAACTTGAAGCTCAGGTAAGGGGCTAGTAGATGCAATGAATGACGATGAAGATAGAGCCCAACTGAAG |
| ADH1t-HXT7p-F | AGCCTTCAGTTGGGCTCTATCTTCATCGTCATTCATTGCATCTACTAGCCCCTTACCTGAGCTTCAAGTTTTTGATTAAAATTAAAAAAACTTTTTGTTTTTGTGTTTATTCTTTGTTCTTAGAAAAGAC |
| FBA1p-ADH2t-R | TCGACGTATGCAACGTATGTGAAGCCAAGTAGGCAATTATTTAGTACTGTCAGTATTGTTATCAGTGCATCATTTGTGAGGGTTCAATAATTGAAATTATAGGGTGGACGTCAAGAC |
| ADH2t-FBA1p-F | GTCTTGACGTCCACCCTATAATTTCAATTATTGAACCCTCACAAATGATGCACTGATAACAATACTGACAGTACTAAATAATTGCCTACTTGGCTTCACATACGTTGCATACGTC |
| CDC60p-CYC1t-R | TCATCTATATTTCAGCCCCAAAAGATTGTGGTTTCAAGTGAAAATAACTGTCCTACTAATGGAATGTGAGGGAACAACAAAAGTCCTTTACGCATATTTTGTCCCAAAAGAACCAAG |
| CYC1t-CDC60p-F | TCTTGGTTCTTTTGGGACAAAATATGCGTAAAGGACTTTTGTTGTTCCCTCACATTCCATTAGTAGGACAGTTATTTTCACTTGAAACCACAATCTTTTGGGGCTGAAATATAGATGAG |
| TEF1p-TDH1t-R | TGCGCGGAGTCCGAGAAAATCTGGAAGAGTAAAAAAGGAGTAGAAACATTTTGAAGCTATCTGGACTAAAAGTGGAGCCGGAATAGAGACTCTCTTAGTATCAGTATCCACCTCTTC |
| DBOX-CDC60p-R | AGCTTTTTAGATGAATCTGCCATTATAGACTGGTTAAAGATAT |
| CDD60p-DBOX-F | ATATCTTTAACCAGTCTATAATGGCAGATTCATCTAAAAAGCT |
| TDH1t-DBOX-R | TCCTCATCAAGATTGCTTTATTTAGAATGGAGTAGTAACTTG |
| DBOX-TDH1t-F | AGTTACTACTCCATTCTAAATAAAGCAATCTTGATGAG |
| TDH1t-TEF1p-F | TGAAGAGGTGGATACTGATACTAAGAGAGTCTCTATTCCGGCTCCACTTTTAGTCCAGATAGCTTCAAAATGTTTCTACTCCTTTTTTACTCTTCCAGATTTTCTCGGACTCCGCGCATC |
| CYC1t-TEF1p-F | ATGCGTAAAGGACTTTTGTTGTTCCCTCACATTCCA ATAGCTTCAAAATGTTTCTACTCCTTTTTTACTCTTCCAG |
| TEF1p-CYC1t-R | ATCTGGAAGAGTAAAAAAGGAGTAGAAACATTTTGAAGCTATTGGAATGTGAGGGAACAACAAAAGTCCTTTACGCATATTTTG |
| TYB-Z3-R | AGCGGTCATCACTGTATAAATTGCAAGTATGTACCAGAAGCACGTGAAGTGAAAAAGGCAAAAGACAAAGGCGAAAAATTGGGCATTACGCCCGAAGGTTTGCCAGTTAAAGGAGCGATGGCTTCCCCGCTATACTTAAG |
| Construction of gene expression cassettes A | |
| TYB-Z5-F | ACACAGTATATTATAATATACCAATAACACGAACTCCAGTCAGTAGATCGTTGCCGATCTTCATTCTAACAAGACAAGAGATGAAAGTATTACAACAGAAAGATGGCACTAAAGATGCCGATTTGGGCGCGAATC |
| TEF1t-PYK2P-F | ATCTCTCTTTTACGTAAAGCCTGCGAGATCCTCTTAAGTATAGCGGGGAAGCCATCGCTTTTATGAACATATTCCGATATCTCTCACGATACTAATATTCG |
| PYK2P-TEF1t-R | TCGAATATTAGTATCGTGAGAGATATCGGAATATGTTCATAAAAGCGATGGCTTCCCCGCTATACTTAAGAGGATCTCGCAGGCTTTACGTAAAAGAG |
| CYC1t-PGK1p-F | TCTTTTGGGACAAAATATGCGTAAAGGACTTTTGTTGTTCCCTCACATTCCATATTTTAGATTCCTGACTTCAACTCAAGACGCACAGATATTATAACATCTG |
| PGK1p-CYC1t-R | AGATGTTATAATATCTGTGCGTCTTGAGTTGAAGTCAGGAATCTAAAATATGGAATGTGAGGGAACAACAAAAGTCCTTTACGCATATTTTGTCCCAAAAG |
| TYB-Z5-R | TCGATGTACTTTTCTTTTTAAAATCGAATTATCAGCGATTATTCAGCCGGCTAAGAAGATGTTCTTATTGTTCTTATGCTTCAAAAATAAAGCATATCCAGGACTAATACATCCATTTTGTTGCAAGTGGGATGAGCTTG |
| TYB-Z6-F | AGTGTTGCGTAGGCACTTCGAACAAGTAGTCAGTAATATCACCTTTTAACATCTAATCATCAAAAGAGACATTTTTTGGGATTAATTGTTTATAAAAGCTATGAACTTAGGTCTACAGAATATTTTAGATTCCTGACTTCAACTCAAGACGCACAGATATTATAACATCTG |
| TYB-Z6-R | ATGTTGAATTGCAAGAATTGATGAAGTAATAGGATAAGGAATGACAACGTATAAAATTGAAGGAGAAATAAAAATATATATTATATAGAAGTATCGGGCCCCTTTCGTAGATTCCTATATCCTTTTTGTTGCAAGTGGGATGAGCTTGGAGCAGGAAGAATACACTATACTG |
| TYB-Z7-F | ATTCCCTTTTACGGATTCCTAAATCCTCGAGGAGAACTTCTAGTACATTCTACATACCTAATATTATTGCCTTATTAAAAATGGAATCCCAACAATTATCTCAAAATTCACCCTATTTTAGATTCCTGACTTCAACTCAAGACGCACAGATATTATAACATCTG |
| TYB-Z7-R | ATGTGATGAATTTTGAGAGCCCACTTTTGTTGGGGACGATTTAATAAAATAAAATGATTACACTGGTGGGAAATAAGAATCAACTACTGTATATTGACTAGTATTGATGTTGTTTTTTGTTGCAAGTGGGATGAGCTTGGAGCAGGAAGAATACACTATACTG |
| TYB-Z8-F | ACGCTGTATTGGAGAGATATATTCTAAAATATTGATAATTAATGGCAAAAAGGCAGTATTAATGAAGGTTGAATATGGATCTTAATATAATCGTATAAGAGGGGCATAAAATATACGTATTTTAGATTCCTGACTTCAACTCAAGACGCACAGATATTATAACATCTG |
| TYB-Z8-R | GATCAATATAACAATGCGTCTGGTAATTACCATAACACTTAATGTAGCAACTCGTATAACAAACGTATCATGCTCAATTAATATAACCTTATCTCTGCTTCAGCACTGTCTGAGCTACTCTTTTATTTTGTTGCAAGTGGGATGAGCTTGGAGCAGGAAGAATACACTATACTG |
| TYB-Z9-F | ACTTATATTACTAGTATATTATATACGGTGTGAAAAGACGACATGAAGATTGAGGAAATGGTCCCAAGATCTAATGGACACTGAAATGCAAAAGTTGATAATGTAACATGAAAATTAAGTATTTTAGATTCCTGACTTCAACTCAAGACGCACAGATATTATAACATCTG |
| TYB-Z9-R | ACTGCTGATATTTCATTGTTGAAAAGGCATGATATAAGATGCACAAAGTATATTATAACTTATTATCAGAGATATAGGAACAAAGAAAAAGGGAAATTCATATCTCTATGTAAAAATACCATTATTTTTTTGTTGCAAGTGGGATGAGCTTGGAGCAGGAAGAATACACTATACTG |
| Construction of gene expression cassettes B | |
| TYB-Z10-F | ATTGCTCTTATATGTGACTTTCAGATCTCATTCAATTAATAGACTCTTAACTCAAATTCAGTACTTTCTGACATGCTCATTTACATTTCCTTTTGTAATAGTGCTTTTTTTGATTAAAATTAAAAAAACTTTTTGTTTTTG |
| PGK1t-TEF1P-F | TGCTCCAAGCTCATCCCACTTGCAACAAAAATAGCTTCAAAATGTTTCTACTCCTTTTTTAC |
| TEF1P-PGK1t-R | AGTAAAAAAGGAGTAGAAACATTTTGAAGCTATTTTTGTTGCAAGTGGGATGAGCTTGGAGC |
| ADH2t-PGK1p-F | TCAATTATTGAACCCTCACAAATGATGCACTGACTGTAATTGCTTTTAGTTGTGTATTTTTAG |
| PGK1p-ADH2t-R | ACTAAAAATACACAACTAAAAGCAATTACAGTCAGTGCATCATTTGTGAGGGTTCAATAATTG |
| TYB-Z10-R | AGAATATTATGTGGCGTATCAATGCCAAAGTATTATATAATGTGTTGAAACGAGCGGTATTTAATAGTAACATGAGTTACTATGGTAACGATCTAAATGTTTATGGCTTCCCCGCTATACTTAAGAGGATCTCGCAGGCTTTAC |
| TYB-Z11-F | AGGCATGATATTAGATGCACAAAGTATATTATAACTTATGATCAGAGATATAGGAACAAAAAAAAGGGAAAATTCATATCTATATGTGAAAATACCATTATTTCCTCTTTTTTGATTAAAATTAAAAAAACTTTTTGTTTTTG |
| TYB-Z11-R | AGTACTTATAGTACCAGTATATTATATACGGTGTGAAAAGACGACATGAAGATTGAGAAAATGGTCCCAAGATTTAATGGACACTGAAATGCAAAAGTTGATAATGTAATATGAAAAATGGCTTCCCCGCTATACTTAAGAGGATCTCGCAGGCTTTAC |
| TYB-Z12-F | ACGCCAAAAGTAGTTACCAGCACAGATAAACCCTTGATGCGTGCGTAACCATCGGCGGCATAGGCGGCGTTCAGCTCATTTGCATTACCAGCCCATCTCAATCCATCTACCTTTTTTTGATTAAAATTAAAAAAACTTTTTGTTTTTG |
| TYB-Z12-R | ATATAGCAAAAACATATTGCCAACAAAATGTCTGAAATTACTCTTGGAAAATACTTATTTGAAAGATTGAAGCAAGTTAATGTTAACACCATTTTTGGGCTACCAGGCGACTTCAACTTGTCATGGCTTCCCCGCTATACTTAAGAGGATCTCGCAGGCTTTAC |
| TYB-Z13-F | AGCACAATAATACCGTGTAGAGTTCTGTATTGTTCTTCTTAGTGCTTGTATATGCTCATCCCGACCTTCCATTTTTTTTTTCTTGGAATCAGTACATAGCAGGTATGAGTTTTTTTTGATTAAAATTAAAAAAACTTTTTGTTTTTG |
| TYB-Z13-R | ATCCTATATGCATAATATTATAGTCTTTGCCAACAATCGAAACCAAACATATATCTTAAAATACACCACTTTCTCAAATAAATTCGTTAAATAACGGTGTGTTGAAATGTTTAATGGCTTCCCCGCTATACTTAAGAGGATCTCGCAGGCTTTAC |
| Construction of gene expression cassettes HEM2+HEM3 | |
| TYB-Z14-F | AGTTGACAATTTAATAGAATGGATTAATCGTAATTTTCAGAAACGTAGAAAAAGAAAAACAATTAAAACATTATATTAAGATTATTGATTTGCCTTTTAAGGGTCCATACTATAGCTTCAAAATGTTTCTACTCCTTTTTTACTCTTCCAG |
| TYB-Z14-R | ATTCCTATAACGTCGGGAGAATTGCTTGTATACTATGTATACCTAATGTTATAGGATTCGGCAACAATGCAATCCGAACAATTATCAAATTACTCGCCAGTTTCTCAGGTATATATATTGGAATGTGAGGGAACAACAAAAGTCCTTTAC |
| TEF1p-HEM3-F | TCTAATCTAAGTTTTAATTACAAAATGGGCCCTGAAACTCTACATATTG |
| TEF1t-HEM3-R | ACTAGAAAAGTCTTATCAATCTCCTCATTTGATTCTGTCTAAATTAATTTC |
| HEM3-TEF1p-R | ACCAATATGTAGAGTTTCAGGGCCCATTTTGTAATTAAAACTTAG |
| HEM3-TEF1t-F | TGAAATTAATTTAGACAGAATCAAATGAGGAGATTGATAAGACTTTTCTAG |
| TEF1p-HEM2-F | TCTAATCTAAGTTTTAATTACAAAATGCATACAGCTGAATTTTTGGAAAC |
| CYC1t-HEM2-R | TCGACAAAGGAAAAGGGGCCTGTTTAGTTTTCTTCATCTAACCAG |
| HEM2-TEF1p-R | TCCAAAAATTCAGCTGTATGCATTTTGTAATTAAAACTTAGATTAG |
| HEM2-CYC1t-F | ACTGGTTAGATGAAGAAAACTAAACAGGCCCCTTTTCCTTTGTCG |
| Construction of gene expression cassette HEM12 | |
| THS1p-HEM12-F | AGCTAACAGAGCAAGAAAATAAAACGGAGATGGGTAACTTTCCAGCTCCAAAAAACG |
| HEM12-THS1p-R | TCGTTTTTTGGAGCTGGAAAGTTACCCATCTCCGTTTTATTTTCTTGCTCTGTTAGC |
| SSD1t-HEM12t-R | ACAGCAATGACGATATTGGTAGAAGTTACTTCGAACCAATTCTGTGGCACTC |
| HEM12-SSD1t-F | AGTGCCACAGAATTGGTTCGAAGTAACTTCTACCAATATCGTCATTGCTGTTTTTC |
| TYB-Z15-F | ATGGTGATAATGATTGTGATTTTATTTGTTGGAATAACAATCAACTATCATCCATTCACTGGTATCAATATTACTGGTATATTATCTTATATGGTGTAGGAAGATGGCATAATATAATATCTTAGCTCCGTGGTCCGCTTCCTTTG |
| TYB-Z15-R | AGTCCACAAAGGACACAAATAATTTTACATAATAACGATATTACCTCTTTTTCCATTTTATGCGTTGTCGTTCACTATCCTGTTACATTATCAATCTTCGCATTTTAGCTTTTACCA CGACCTCAAATGTTTACGCAGTAGTTTCTGAAGTAC |
| Construction of ROX1 gene knockout | |
| ROX1-TYBS-F | ACATGTCCCTTGAGCCCCTCTGTTC |
| TYBS-ROX1-R | TGGAATCTTAGGTGTAGAGGATTTAGGATTC |
| ROX1-TYBX-R | ACATGTCCCTTGAGCCCCTCTGTTC |
| TYBX-ROX1-F | ATTATAAACTCAAGCAACACTGAGGTCACCG |
| Construction of HMX1 gene knockout | |
| TYBS-HMX1-F | AGTGGGTGAGGGTATGATTGTATTGCTACTGTCCTC |
| TYBX-HMX1-R | ATTATCGCGATCTGGGTTCTTTACTTCTTG |
| HMX1-TYBS-F | AGCCTGTTGTGTGGCGACTTCCTTAG |
| HMX1-TYBX-R | TGTGAAGGTCAAACTAGAATGTAAGCGTATTC |
| Construction of gene expression cassette ZWF1 | |
| TEF1p-ZWF1-F | ATCTAATCTAAGTTTTAATTACAAAATGAGTGAAGGCCCCGTCAAATTCG |
| ZWF1-TEF1p-R | TCGAATTTGACGGGGCCTTCACTCATTTTGTAATTAAAACTTAGATTAGATTG |
| ZWF1-TEF1t-F | AGAAGATACGAAGGATAATTAGGGAGATTGATAAGACTTTTCTAG |
| TEF1t-ZWF1-R | ACTAGAAAAGTCTTATCAATCTCCCTAATTATCCTTCGTATCTTCTG |
| TYB-Z18-R | ATTTCCTTCAAAAGTAGTTGCTTCTTCTTAGACTTTCTTACCGGCTTGTATTTGTATTCAGGATACTTCCTTTCATGTTCTAGTTTCTCCTTCTCCGCTAGATTTTATAGCTTCAAAATGTTTCTACTCCTTTTTTACTCTTCCAG |
| TYB-Z18-F | ATTCTGTTCAGACAGCACTACCACAGGATCTTAATAGACGAATGGACCGCTCAAGGTGTGGAAATACCCCATAATTCAAACATTTCTAAAATTATTGGTACGAAGTGGAAGGGCTTACAATGGCTTCCCCGCTATACTTAAGAGGATCTCGCAGG |
| Construction of gene expression cassette GND1 | |
| TYS1p-GDN1-F | ATAACCGCATACTCTAATTGACGATAACATGTCTGCTGATTTCGGTTTGATTG |
| GDN1-TYS1p-R | ACCAATCAAACCGAAATCAGCAGACATGTTATCGTCAATTAGAGTATGCGGTTATG |
| GDN1-TPS1t-F | ATGTTTCTTCCTCTACATACCAAGCTTAATGAACCCGATGCAAATGAGACGATC |
| TPS1t-GDN1-R | ATCGTCTCATTTGCATCGGGTTCATTAAGCTTGGTATGTAGAGGAAGAAAC |
| TYB-Z19-R | ACCTCCTCATACTCTCTTGCTTAGTCTAAGGAGGAGCTATTTAACAGTGCACAATATAACACAGCATATATATATATACACACACACATAAAATAACCGCAAAACTTTGGACCGTCAGAGTCGTTCTTACCTCTTC |
| TYB-Z19-F | ACCTCTCACATGTTTTTCTCTGCTGTTTTTCCTTCCCTATTCTTCATATTTTGATATTATTTCATGTATATATTATGTTTGTATTTAGATTTTTTTTTTTATACGCCTTGCGCTTACTCGAATAG GCCTCCCTAG CTATTC |
| Construction of gene expression cassettes TKL1+TKL2+TAL1 | |
| ALA1p-TKL1-F | AGCAATTAACTACATCAACTAGAACCATAATGACTCAATTCACTGACATTGATAAGC |
| TKL1-ALA1p-R | AGCTTATCAATGTCAGTGAATTGAGTCATTATGGTTCTAGTTGATGTAGTTAATTGC |
| TKL1-PYC2t-F | AATTTCTCCTTTGAAAAAAGCTTTCTAACTCGTTAATTATATTTTATGACATCTG |
| PYC2t-TKL1-R | TCAGATGTCATAAAATATAATTAACGAGTTAGAAAGCTTTTTTCAAAGGAGAAATTAG |
| PYC2t-MSY1p-F | TGCTGCGGACTTGGCGCAAACGAATTATTATATCTTAATATTGGACTTGAAGCTG |
| MSY1p-PYC2T-R | ACAGCTTCAAGTCCAATATTAAGATATAATAATTCGTTTGCGCCAAGTCCGCAGC |
| MSY1p-TKL2-F | AGCAATCTACAGGTAGACATTGTGAATCATGGCACAGTTCTCCGACATTGATAAAC |
| TKL2-MSY1p-R | AGTTTATCAATGTCGGAGAACTGTGCCATGATTCACAATGTCTACCTGTAGATTGCTG |
| APE2t-TKL2-R | ATACTTGTTGACGACATCACGGTCTCTGTTAGAAAGCTCTTCCCATAGGAGAAAGC |
| TKL2-APE2t-F | TGCTTTCTCCTATGGGAAGAGCTTTCTAACAGAGACCGTGATGTCGTCAACAAGTATTTG |
| APE2t-FRS1p-F | ATGTTAGTATCCAATAAATGCAGCGCACTTGATCTCTTCTGTTTATTTGTGAAAG |
| FRS1p-APE2t-R | AGCTTTCACAAATAAACAGAAGAGATCAAGTGCGCTGCATTTATTGGATACTAACATAC |
| FRS1p-TAL1-F | ACACACATCAATAACACCGAGCCAGCATGTCTGAACCAGCTCAAAAGAAAC |
| TAL1-FRS1p-R | TGTTTCTTTTGAGCTGGTTCAGACATGCTGGCTCGGTGTTATTGATGTGTG |
| TAL1-APT15t-F | ACTTGATTGAAAAGAAAGTTACCGCTTAATTTAACGCTTCCTGGGAACTGCAGC |
| APT15t-TAL1-R | AGCTGCAGTTCCCAGGAAGCGTTAAATTAAGCGGTAACTTTCTTTTCAATCAAG |
| TYB-Z19-F | ACCTTAGCATCCCTTCCCTTTGCAAATAGTCCTCTTCCAACAATAATAATGTCAGATCCTGTAGAGACCACATCATCCACGGTTCTATACTGTTGACCCAATGCGTCTTTAGTAGGACAGTTATTTTCACTTGAAACCAC |
| TYB-Z19-R | ACTAAGGGTACTGTTGACATTGCGAAGAGCGACAAAGATTTTGTTATCGGCTTTATTGCTCAAAGAGACATGGGTGGAAGAGATGAAGGTTACGATTGGTTGATTATGACACCCGAGAGGCTGAAGGCAGAGAAGTTTCTGGAACTG |
| Construction of GPP1 gene knockout and gene expression cassettes LmXFPK+CkPTA | |
| GPP1-TYBX-R | ACTTCAGTTTCTAAGTGGAAGACCTTGGTC |
| GPP1-TYBS-F | ACACACCCTGGTCAATCGTACCAAGGGATATC |
| GPP1-TYS1p-TYBS-R | AGGGAGGCCTATTCGAGTAAGCGCAAGGTGCGATGGTTTGTATATTTGCTTTTGTTG |
| GPP1-ATP15t-TYBX-F | AGAAACTTCTCTGCCTTCAGCCTCTCTTTTCTTTTATTTTTTTGATAAAACTACTAC |
| GPP1-TYBS-TYS1p-F | AGTTTTATCAAAAAAATAAAAGAAAAGAGAGGCTGAAGGCAGAGAAGTTTCTGG |
| GPP1-TYBX-ATP15t-R | ATAACCGCATACTCTAATTGACGATAACATGTCTGCTGATTTCGGTTTGATTG |
| CDC60p-LmXFPK-F | AGAATATAAAAAAAATATCTTTAACCAGTCTATAATGGCTGACTTTGACTCTAAGGAG |
| LmXFPK-CDC60p-R | ACTCCTTAGAGTCAAAGTCAGCCATTATAGACTGGTTAAAGATATTTTTTTTATATTC |
| LmXFPK-ATP15t-F | AGACTGGACCTGGTCTCCACTGAAGTAATTTAACGCTTCCTGGGAACTGCAGCTC |
| ATP15t-LmXFPK-R | AGCTGCAGTTCCCAGGAAGCGTTAAATTACTTCAGTGGAGACCAGGTCCAGTCTG |
| ATP15t-THS1p-F | AGAAACTTCTCTGCCTTCAGCCTCTCAATATCTTAGCTCCGTGGTCCGCTTCC |
| THS1p-ATP15t-R | AGGAAGCGGACCACGGAGCTAAGATATTGAGAGGCTGAAGGCAGAGAAGTTTCTG |
| THS1p-CkPTA-F | AGCTAACAGAGCAAGAAAATAAAACGGAGATGAAATTGATGGAAAATATTTTTGGTTTG |
| CkPTA-THS1p-R | AACCAAAAATATTTTCCATCAATTTCATCTCCGTTTTATTTTCTTGCTCTGTTAGC |
| CkPTA-SSD1t-F | ACCGCTGTTCAAGCTCAAGCTCAAGGTTAACTTCTACCAATATCGTCATTGCTGTTTTTC |
| SSD1t-CkPTA-R | AGAAAAACAGCAATGACGATATTGGTAGAAGTTAACCTTGAGCTTGAGCTTGAACAGCGG |
| Construction of gene expression cassettes for transporters | |
| TEF1p-MtABCG10-F | TCTAATCTAAGTTTTAATTACAAAATGGAAGGTACTGATATCTATAG |
| MtABCG10-TEF1p-R | TCTATAGATATCAGTACCTTCCATTTTGTAATTAAAACTTAGATTAG |
| MtABCG10-TEF1t-F | TCTTTCAACTTCCAAAAGAGATGAGGAGATTGATAAGACTTTTCTAG |
| TEF1t-MtABCG10-R | ACTAGAAAAGTCTTATCAATCTCCTCATCTCTTTTGGAAGTTGAAAG |
| TEF1p-McoABC-F | TCTAATCTAAGTTTTAATTACAAAATGTGGTCCTCTTCTGAAACTGCTTTCAC |
| McoABC10-TEF1p-R | AGTGAAAGCAGTTTCAGAAGAGGACCACATTTTGTAATTAAAACTTAGATTAG |
| McoABC10-TEF1t-F | AGTCTTTCAACTTCCAAAGAAGATAAGGAGATTGATAAGACTTTTCTAG |
| TEF1t-McoABC-R | ACTAGAAAAGTCTTATCAATCTCCTTATCTTCTTTGGAAGTTGAAAGACTTAAC |
| TEF1p-CrTPT2-F | TCTAATCTAAGTTTAATTACAAAATGTTGTTGAACTCTTCTGCTTCTGCTAAC |
| CrTPT2-TEF1p-R | AGCAGAAGCAGAAGAGTTCAACAACATTTTGTAATTAAAACTTAGATTAG |
| CrTPT2-TEF1t-F | AGGCTTTCAACTTCCAAAGAAGATAAGGAGATTGATAAGACTTTTCTAG |
| TEF1t-CrTPT2-R | ACTAGAAAAGTCTTATCAATCTCCTTATCTTCTTTGGAAGTTGAAAGCCTTG |
| TEF1p-CjABCB2-F | TCTAATCTAAGTTTAATTACAAAATGGCTGAAGAAAACGGTTTCAACGGTG |
| CjABCB2-TEF1p-R | TCACCGTTGAAACCGTTTTCTTCAGCCATTTTGTAATTAAAACTTAGATTAG |
| CjABCB2-TEF1t-F | TGGTTGCTTTGCACATGACTGCTTCTTAAGGAGATTGATAAGACTTTTCTAG |
| TEF1t-CjABCB2-R | ACTAGAAAAGTCTTATCAATCTCCTTAAGAAGCAGTCATGTGCAAAGCAACCAAAG |
| TYB-Z22-F | ATGGTTGATGGTGTCATGAAGAATGAACAAAGAGCCCTAAGACGTATCGCAAAGAAGCATCACAAGAAAAAGTAGTAACAAAGAGCATTATTTTTCCATTCCCATAGCTTCAAAATGTTTCTACTCCTTTTTTACTCTTCCAG |
| TYB-Z22-R | ACTGTGCACTCGTATTTTCTGAATTCAAAACGATAGGTTTCCTATAAGTTTTTTGATGATAATTTGATTAATTCTATGTATACATAATATATCTATTGCTTTCTTTTATGGCTTCCCCGCTATACTTAAGAGGATCTCGCAG |
| Construction of gRNA plasmids | |
| gRNA-Z3-F | CCAGAGTGTATAAAATGTGGGTTTTAGAGCTAGAAATAGC |
| gRNA-Z3-R | CCACATTTTATACACTCTGGGATCATTTATCTTTCACTGC |
| gRNA-Z5-F | TGTACTTATTAAAGTAGAAGGTTTTAGAGCTAGAAATAGCAAG |
| gRNA-Z5-R | CTTCTACTTTAATAAGTACAGATCATTTATCTTTCACTGCGGAG |
| gRNA-Z6-F | TGTACCAAAAGTTATCCTGTGTTTTAGAGCTAGAAATAGCAAG |
| gRNA-Z6-R | ACAGGATAACTTTTGGTACAGATCATTTATCTTTCACTGCGGAG |
| gRNA-Z7-F | ATAGAATTACTATTGAAGAGGTTTTAGAGCTAGAAATAGCAAG |
| gRNA-Z7-R | CTCTTCAATAGTAATTCTATGATCATTTATCTTTCACTGCGGAG |
| gRNA-Z8-F | ATAATTAATGTTGAACCAATGTTTTAGAGCTAGAAATAGCAAG |
| gRNA-Z8-R | ATTGGTTCAACATTAATTATGATCATTTATCTTTCACTGCGGAG |
| gRNA-Z9-F | GACAGCATATTAAACAGAAGGTTTTAGAGCTAGAAATAGCAAG |
| gRNA-Z9-R | CTTCTGTTTAATATGCTGTCGATCATTTATCTTTCACTGCGGAG |
| gRNA-Z10-F | AAATCCTATACGATGAAGTAGTTTTAGAGCTAGAAATAGCAAG |
| gRNA-Z10-R | TACTTCATCGTATAGGATTTGATCATTTATCTTTCACTGCGGAG |
| gRNA-Z11-F | TTAAGGACAGAATATTAAACGTTTTAGAGCTAGAAATAGCAAG |
| gRNA-Z11-R | GTTTAATATTCTGTCCTTAAGATCATTTATCTTTCACTGCGGAG |
| gRNA-Z12-F | CCTATTGGACAAGATTTACGGTTTTAGAGCTAGAAATAGCAAG |
| gRNA-Z12-R | CGTAAATCTTGTCCAATAGGGATCATTTATCTTTCACTGCGGAG |
| gRNA-Z13-F | CATCCACGAAAACATACACAGTTTTAGAGCTAGAAATAGCAAG |
| gRNA-Z13-R | TGTGTATGTTTTCGTGGATGGATCATTTATCTTTCACTGCGGAG |
| gRNA-Z14-F | TATCCTGAATGTTCTCTCCCGTTTTAGAGCTAGAAATAGCAAG |
| gRNA-Z14-R | GGGAGAGAACATTCAGGATAGATCATTTATCTTTCACTGCGGAG |
| gRNA-Z15-F | TGAGAAACGGCTATCGGATGGTTTTAGAGCTAGAAATAGCAAG |
| gRNA-Z15-R | CATCCGATAGCCGTTTCTCAGATCATTTATCTTTCACTGCGGAG |
| gRNA-Z16-F | GGTGTGGGTTTAGATGACAAGTTTTAGAGCTAGAAATAGCAAG |
| gRNA-Z16-R | TTGTCATCTAAACCCACACCGATCATTTATCTTTCACTGCGGAG |
| gRNA-Z17-F | ACCGGAAGATAAGGCACACTGTTTTAGAGCTAGAAATAGCAAG |
| gRNA-Z17-R | AGTGTGCCTTATCTTCCGGTGATCATTTATCTTTCACTGCGGAG |
| gRNA-Z19-F | CATCCACGAAAACATACACAGTTTTAGAGCTAGAAATAGCAAG |
| gRNA-Z19-R | TGTGTATGTTTTCGTGGATGGATCATTTATCTTTCACTGCGGAG |
| gRNA-Z20-F | CTTAAAGGGTAGAAACGGTTGTTTTAGAGCTAGAAATAGCAAG |
| gRNA-Z20-R | AACCGTTTCTACCCTTTAAGGATCATTTATCTTTCACTGCGGAG |
| gRNA-Z22-F | TTGTATAATATTGAAAATAAAGTTTTAGAGCTAGAAATAGCAAG |
| gRNA-Z22-R | TTTATTTTCAATATTATACAAGATCATTTATCTTTCACTGCGGAG |


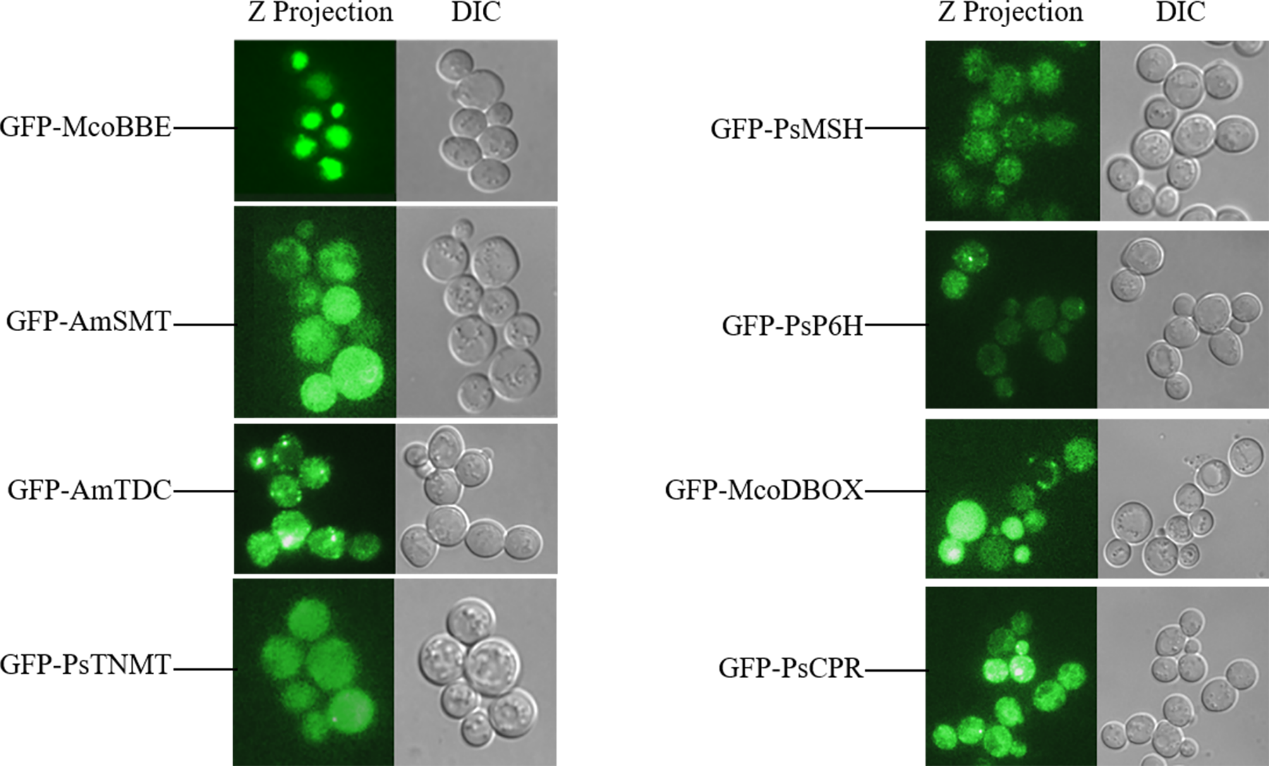


**Fig. S1.** **Expression of fusion proteins.** Target proteins (McoBBE, AmSMT, AmTDC, PsTNMT, PsMSH, PsP6H, McoDBOX, PsCPR) were N-terminally fused with the carboxyl terminal of green fluorescent protein to obtain fusion protein GFP-McoBBE, GFP-AmSMT, GFP-AmTDC, GFP-PsTNMT, GFP-PsMSH, GFP-PsP6H, GFP-McoDBOX and GFP-PsCPR). We obtained recombinant strains (Z0(GFP-McoBBE), Z0(GFP-AmSMT), Z0(GFP-AmTDC), Z0(GFP-PsTNMT), Z0(GFP-PsMSH), Z0(GFP-PsP6H), Z0(GFP-McoDBOX) and Z0(GFP-PsCPR)) through plasmid transformation in Z0. Recombinant strains were cultured for 18 h at 30 °C, centrifuged and resuspended, and collected images on an inverted fluorescence microscope. DIC, differential interference contrast.


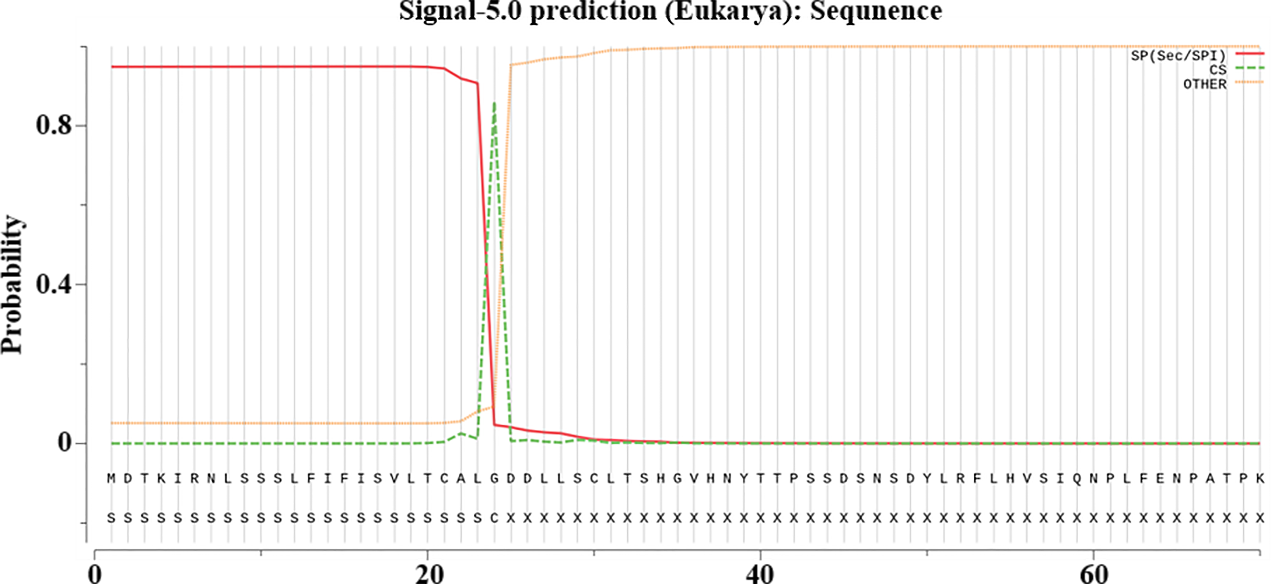


**Fig. S2. The result signal peptide prediction of McoBBE.** The signal peptide of McoBBE was predicted by signalP-5.0 (<https://services.healthtech.dtu.dk/services/>

SignalP-5.0/). The results show that the cleavage site was between pos. 24 and 25: ALG-DD.


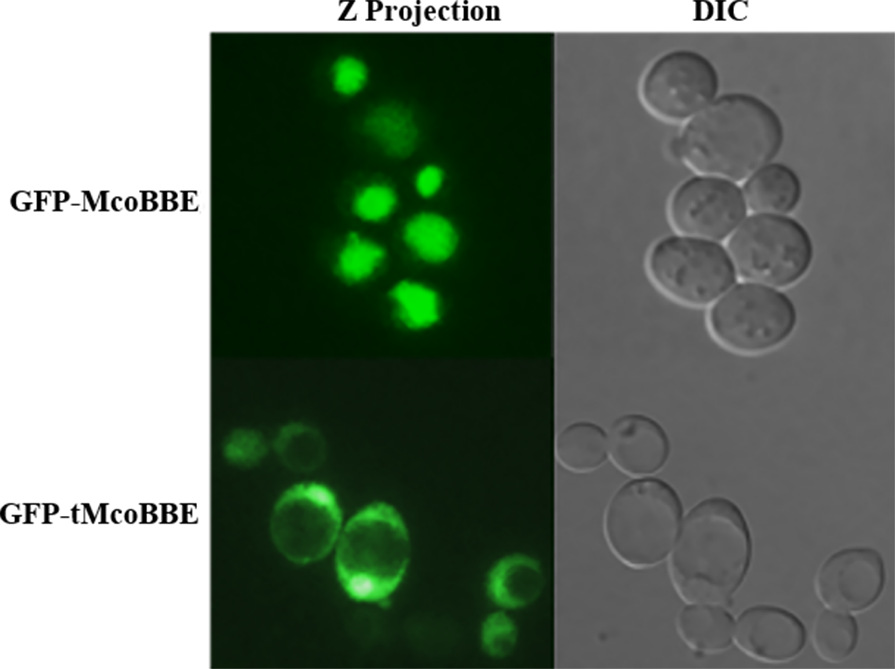


**Fig. S3. Inverted fluorescence microscope analysis of the localization of the mutant tMcoBBE variant expressed in yeast.** GFP-McoBBE and GFP-tMcoBBE are fusion proteins in which the amino terminal of McoBBE or tMcoBBE is fused to the carboxyl terminal of GFP. The engineered strain containing GFP-McoBBE or GFP-tMcoBBE was cultured for 18 h at 30 °C, centrifuged and resuspended, and collected images on an inverted fluorescence microscope. DIC, differential interference contrast.


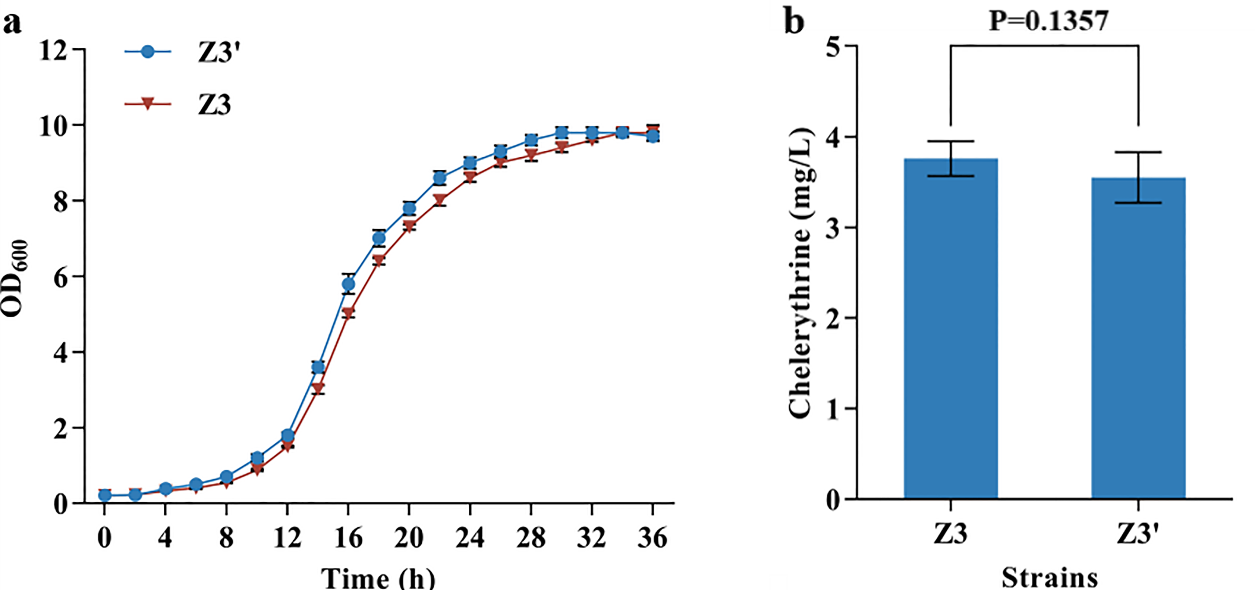


**Fig. S4. Growth profiles and production of chelerythrine of the recombinant strain Z3 and Z3****′. a** Growth profiles of the engineered strains Z3 and Z3′. **b** Production of chelerythrine from (*S*)-canadine in the strain Z3 and Z3′. Statistical analysis was performed by using Student’s t test (two-tailed; two-sample unequal variance; p>0.05, no significant difference).


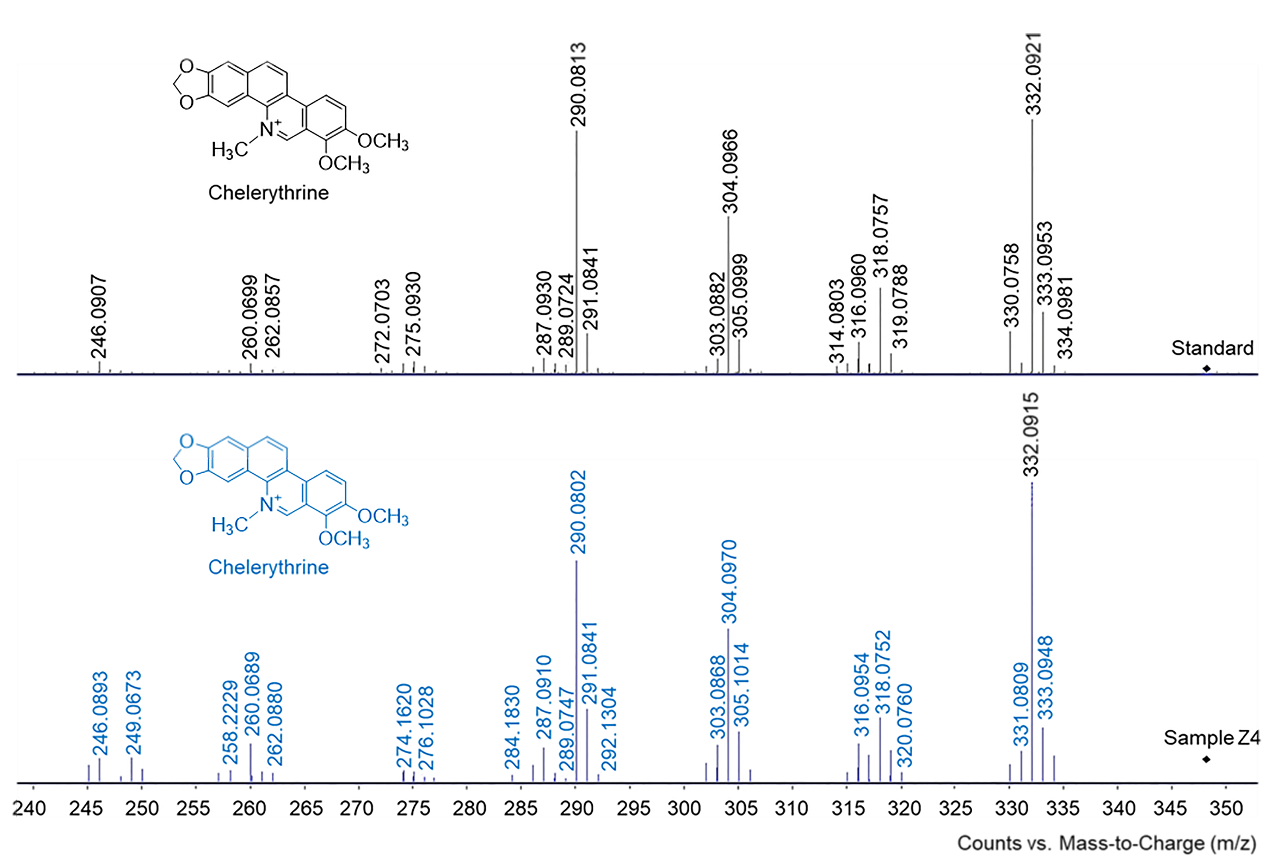


**Fig. S5. LC-MS/MS analysis of the corresponding compound in Z4 and the standard of chelerythrine**. Production ion spectra of chelerythrine (m/z 348.1236) obtained using the positive ion mode at collision energies of 40 eV.


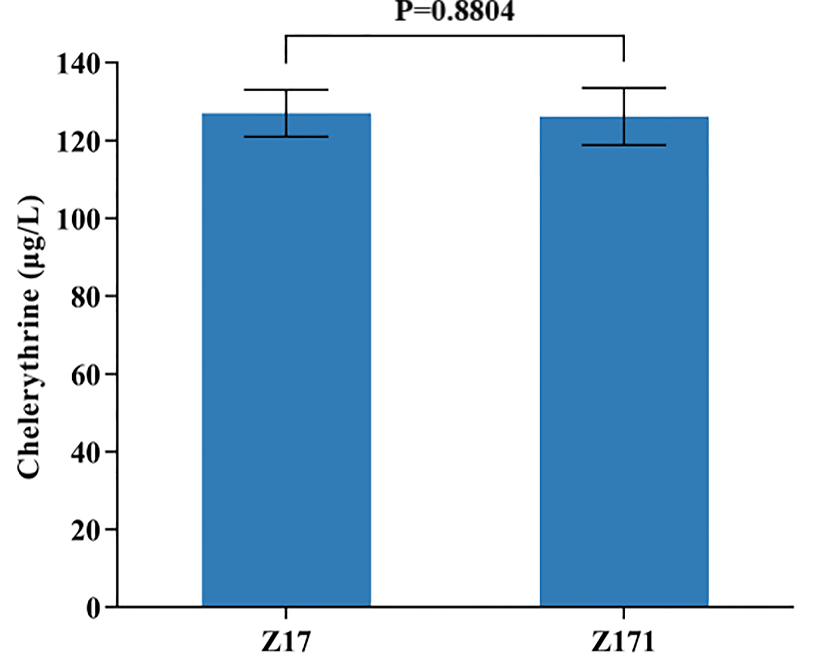


**Fig. S6. Production of chelerythrine in engineered yeast Z17 and Z171**. The *ALD6* gene was overexpressed in Z17 in plasmid form and obtained the engineered strain Z171. Statistical analysis was performed by using Student’s t test (two-tailed; two-sample unequal variance; p>0.05, no significant difference).


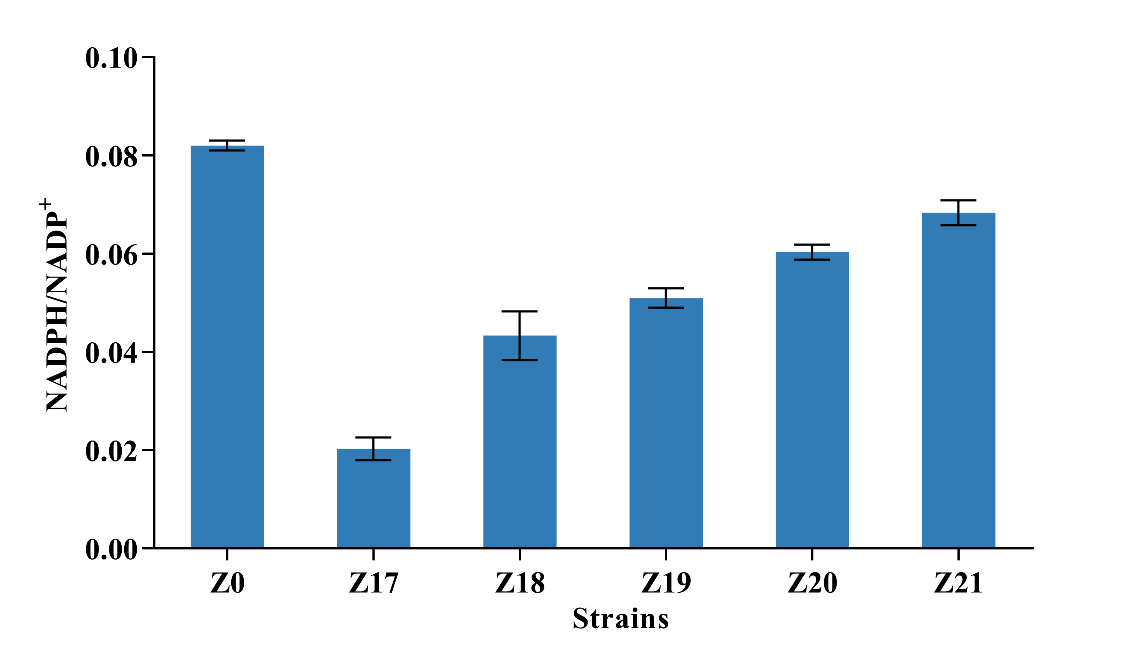


**Fig. S7.** **Cellular NADPH/NADP^+^ ratios in engineered strains**. Measurement of cellular NADPH/NADP+ was conducted using the CheKine NADP+/NADPH assay Kit (catalog no. WST-8; Abbkine).


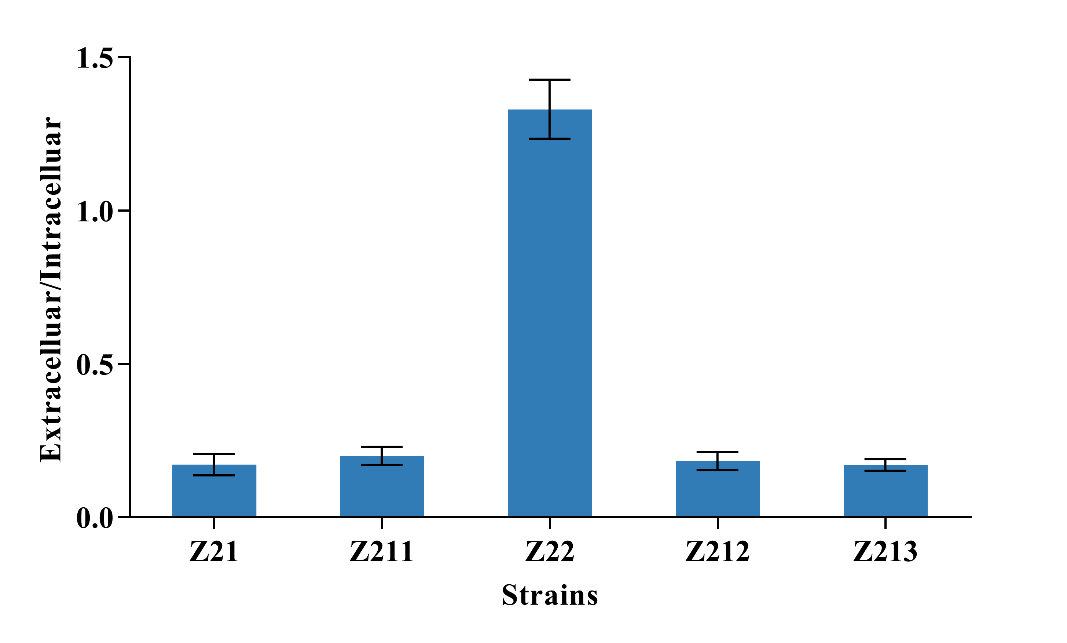


**Fig. S8.** **Intracellular and extracellular distribution of chelerythrine in engineered strains expressing different transporters**. Genes encoding transporters, including *M. cordata* McoABC (*McoABC*), *Medicago truncatula* MtABCG10 (*MtABCG10*), *Catharanthus roseus* CrTPT2 (*CrTPT2*), *Coptis japonica* CjABCB2 (*CjABCB2*), were integrated into strain Z21, respectively, and obtained the engineered strains Z211, Z22, Z212, Z213.


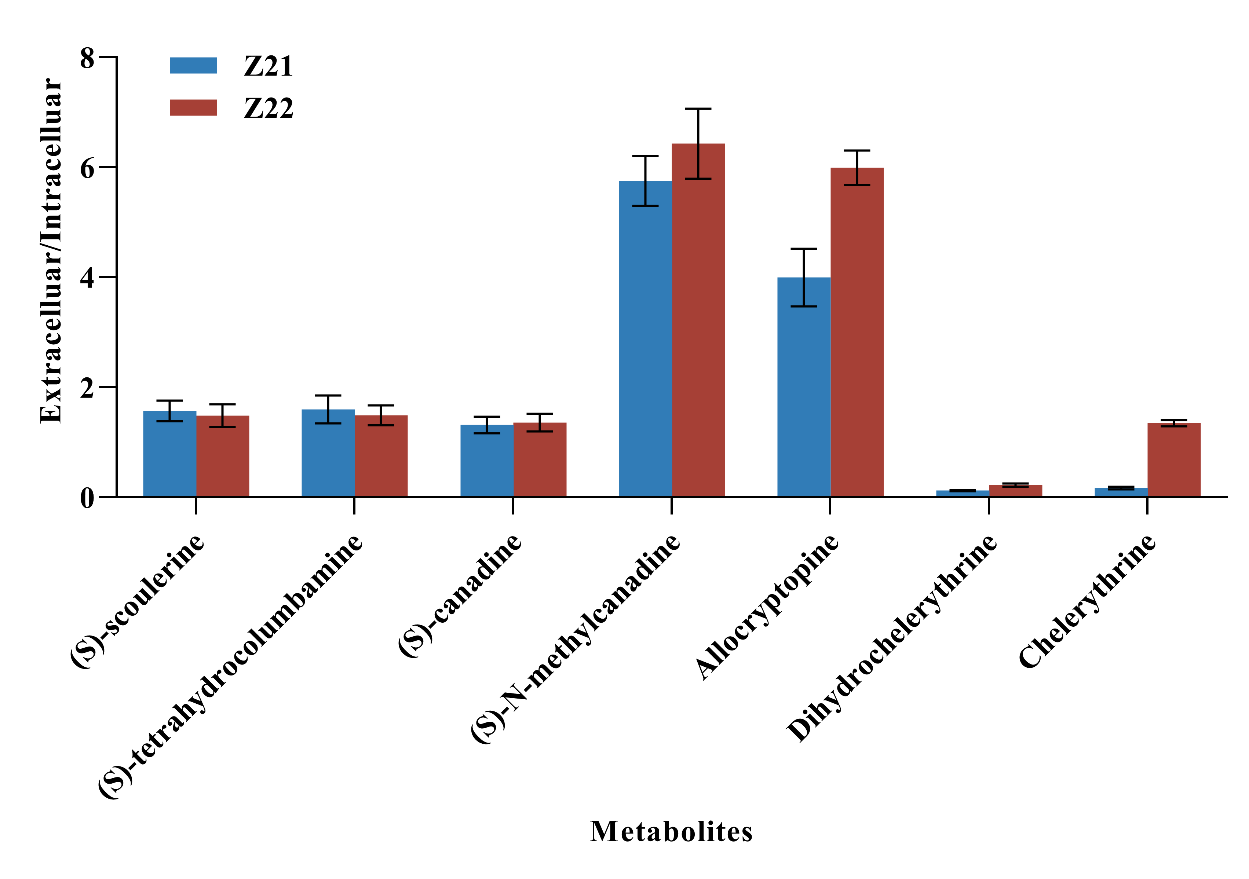


**Fig. S9. Intracellular and extracellular distribution of metabolites in engineered strains**. The ratio of extracellular to intracellular metabolites in recombinant yeast Z21 and Z22.


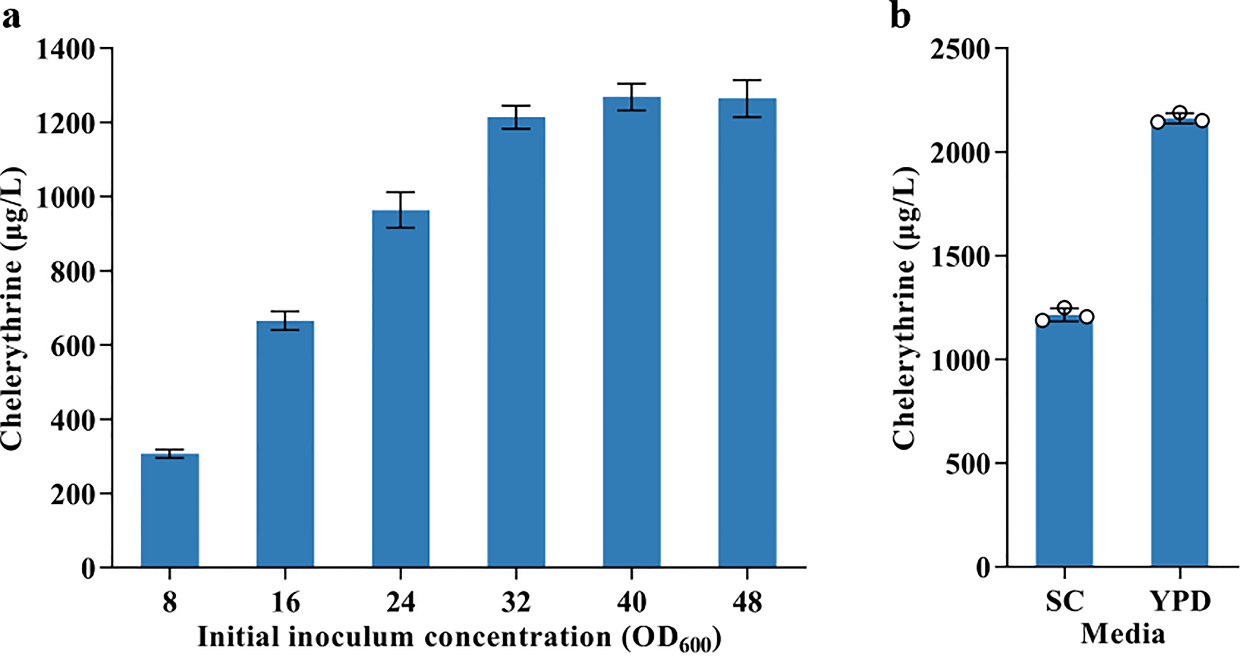


**Fig. S10.** **Fermentation process optimization for chelerythrine production**. **a** Accumulation of chelerythrine in different initial inoculum concentrations of engineered strain Z22. **b** Production of chelerythrine by strain Z22 in different media (SC and YPD medium). OD_600_ of the initial inoculum concentration was 40.
